# Functional Divergence of the N-Lobe and C-Lobe of *Transferrin* Gene in *Pungitius sinensis* (Amur Stickleback)

**DOI:** 10.3390/ani12243458

**Published:** 2022-12-07

**Authors:** Jun Cao

**Affiliations:** School of Life Sciences, Jiangsu University, Zhenjiang 212013, China; cjinfor@163.com

**Keywords:** immune response, iron-binding, antibacterial property, molecular evolution, functional divergence, selection pressure

## Abstract

**Simple Summary:**

As an iron-binding glycosylated protein, transferrin plays key roles in iron metabolism and immune response. Here, a *transferrin* transcript was identified from Amur stickleback, which encoded 679 amino acid peptides harbored obvious N-lobe and C-lobe domains. This *transferrin* was highly expressed in liver and increased by pathogen stimulation. Molecular evolution was performed on the N-lobe and C-lobe domains. Iron-binding capacity and antibacterial characteristics were also compared between N-lobe and C-lobe. These results will have important reference significance for the subsequent functional research.

**Abstract:**

Transferrin is an important iron-binding glycosylated protein and plays key roles in iron-binding and immune response. Here, a 2037-bp open reading frame was obtained from our previous transcriptome sequencing data of Amur stickleback, which encoded a 679 amino acid putative transferrin protein harbored obvious N-lobe and C-lobe domains. The tissue-specific expression pattern showed that the transcript was detected in a variety of tissues, with the highest signal in liver. Moreover, *Streptococcus iniae* pathogen stimulation can increase the expression level of this transcript, implying important immune properties for organisms. Next, N-lobes and C-lobes were obtained from 45 fish species. The phylogenetic tree showed that N-lobes and C-lobes were in two different evolutionary branches, and they had different motif composition. Functional divergence indicated a higher evolutionary rate or site-specific alteration among the N-lobe and C-lobe groups. *Ka*/*Ks* value of C-lobe group was relatively higher than that of N-lobe group, indicating a faster change rate of C-lobe sequences in evolution. Moreover, some sites experiencing positive selection were also found, which may be involved in the iron- or anion-binding, pathogen resistance and diversification of transferrin protein. Differential iron-binding activity was also detected between N-lobe and C-lobe of Amur stickleback transferrin protein with Chrome Azurol S assay. Compared with the C-lobe, the N-lobe showed stronger growth inhibitory activity of *Escherichia coli*, implying their potential antibacterial properties. This study will give a reference for subsequent research of transferrin proteins.

## 1. Introduction

As an essential trace element of an organism, iron functions in many metabolic and physiological processes such as electron transfer, DNA synthesis, etc. [1,2,3]. The bioavailability of iron depends on the Fe^2+^ and Fe^3+^ forms. Among them, the free ferrous iron (Fe^2+^) is highly toxic and can form hydroxyl radicals, causing oxidative damage of macromolecules. Therefore, the free ferric form (Fe^3+^) is necessary for its transportation [4,5]. Fe^3+^ is insoluble, so organisms usually use specialized iron-binding proteins to transport or store it [6]. In addition, excessive iron promotes the production of free radicals and the propagation of pathogens [1,2]. It is thus clear that maintaining an appropriate iron balance is essential for organisms [7]. The maintenance of iron homeostasis requires a series of protein interactions and their regulation, among which transferrin (TF) plays key roles in iron transport and storage [8].

TF is a glycosylated protein with about 70–80 kDa and plays roles in iron metabolism, cell proliferation, inflammation, immune response, and antibacterial functions [9,10]. According to their localizations, structures, and functional characteristics, transferrins are divided into several subclasses including serum transferrin, melanotransferrin, lactoferrin, ovotransferrin, and other transferrin-like proteins [11]. Serum transferrin is mainly responsible for the transport of iron, which is synthesized in the liver [12], while melanotransferrin plays a membrane-bound role present in melanoma [13]. Lactoferrin mainly exists in milk secretion and plays a role in the innate immune [12,14]. Ovotransferrin has antibacterial and antiviral effects, expressed in the oviduct and liver [15]. Structurally, they contain N-lobe and C-lobe. Both domains are composed of highly conserved anion-binding and iron-binding sites [16,17]. They also have similar peptide residues and three-dimensional structure, which are considered to be the result of fusion after ancestral gene duplication [18,19]. Each lobe contains two subdomains which can interact and form ion binding sites. Although the growth of some pathogens is enhanced in the presence of TF, it can limit infections caused by some pathogens through reducing the iron level in the surrounding environment [20,21]. Many reports have confirmed that TF plays important roles in host response to pathogen invasion, and it participates in host innate immunity [22,23]. TF can be cleaved into small fragments by elastase, which can increase the expression of chemokines and cytokines and be used as a key activator of antibacterial response [24,25,26].

TF proteins have undergone a complex evolutionary process. Abundant polymorphisms have been observed in TF of many vertebrates [27]. In mammals, a large number of single nucleotide and/or single amino acid substitutions lead to the TF polymorphisms [28]. In addition, selection pressure also functions in evolution and leads to high DNA polymorphism of fish *TF* genes [29,30,31,32,33]. Furthermore, recombination and gene transformation events also cause the diversification of TF protein alleles [34]. The polymorphism of TF proteins is helpful for organisms to adapt to environmental changes and resist pathogen infection [17,29,31,35].

Transferrin has been studied in some teleost fishes with specific tissue distribution [36,37,38,39,40,41,42,43] and important roles in iron chelation [37], antibacterial reaction [36,37], and immune response [2,23,38,40,43]. The species and resources of fish are extremely rich. As lower vertebrates, although fish do not have some immune organs of higher vertebrates, such as the spinal cord and lymph nodes of mammals, the immune system of fish also consists of the innate and adaptive immune system. Therefore, its research is helpful to understand the evolution of vertebrate immune system. In addition, it also helps to study the species change, environmental adaptation, and evolutionary history and promote the sustainable development of fisheries.

*Pungitius sinensis* (Amur stickleback) is one kind of the Gasterosteidae family. This fish has become an important model due to its small size, strong vitality, and short breeding cycle. Here, one *TF* gene was obtained from Amur stickleback, and its tissue and pathogen induced expression pattern, phylogenetic relationship, motif composition, functional divergence, selection pressure, iron-binding activity, and antibacterial characteristics were analyzed.

## 2. Materials and Methods

### 2.1. Preparation of P. sinensis, RNA Extraction, Expression Patterns in Different Tissues and under Pathogen Infection

All of the procedures complied with and were approved by the Institutional Animal Care and Use Committee (ethical approval number: UJS-LAER-2018120901). The Amur stickleback (*P. sinensis*) was obtained from a farm in Hunan, China. Twenty sticklebacks were cultured in 22–23 °C. Total RNA from six healthy tissues (liver, intestine, kidney, muscle, gill, and heart) was isolated with high-purity Trizol total RNA extraction kit (Sangon, Shanghai, China). RNA quality and concentration were determined with NanoDrop (Thermo Scientific, Wilmington, NC, USA). Next, reverse transcription was performed with M-MLV (TakaRa, Dalian, China) and random primers. The TruSeq Stranded mRNA LTSample prep kit (Illumina, San Diego, CA, USA) was used to construct cDNA. To explore the response of the TF gene to a pathogen, healthy fish was injected with 100 μL of 1 × 10^4^ CFU/mL live *Streptococcus iniae* (Pier and Madin strain) purchased from MingzhouBio, a Gram-positive bacteria and a serious aquaculture pathogen that can infect a variety of aquaculture fish. Each test group was sampled at 3 h, 6 h, 12 h, 24 h, 2 d, 3 d, and 4 d after post-challenge, and liver tissue was collected to extract RNA and reverse transcribe into cDNA as previously described. Quantitative PCR with the LightCycler^®^ 480 II Real-time PCR Instrument (Roche, Basel, Switzerland) was performed using specific primers (Table 1). *Actin* gene was used as an internal reference (Supplementary Data S1). Relative expression level of the *TF* was measured by the 2^−ΔΔCt^ method [44]. Three biological replicates were used for this analysis.

### 2.2. Sequence Retrieval of Fish TFs

To identify potential TF proteins in the 45 fish species, the TF protein sequence of Amur stickleback identified by our previous transcriptome sequencing data [45] was used to BLAST the Ensembl database (http://www.ensembl.org/index.html, accessed on 8 June 2021) [46] with cutoff E-value of 1 × 10^−1^. For each species, the candidate with the highest homology with the Amur stickleback TF protein was selected for subsequent analysis (Supplementary Data S2). Next, Pfam database [47] was used to confirm the candidates based on whether the conserved transferrin domain existed or not and to distinguish sequences of N-lobe and C-lobe.

### 2.3. Phylogeny and Motif Analysis

Multiple sequence alignment of these N-lobes and C-lobes was performed with MUSCLE 3.52 [48]. Subsequently, MEGA v6 [49] was used for phylogenetic analysis using neighbor joining (NJ) method with pairwise-deletion, *p*-distance, and 1000 bootstrap replicates parameters. Conserved motifs were identified with MEME (http://meme-suite.org, accessed on 28 August 2021) [50] in each amino acid sequence with parameters: maximum 6 motif and 6–50 widths.

### 2.4. Functional Divergence Analyses

DIVERGE V2.0 [51] was used to estimate functional divergence among the N-lobes and the C-lobes of fish TF genes. Type I functional divergence can detect specific residue regions, which means that these residues undergo functional change [51]. Type II functional divergence can detect some conserved residues with different biochemical characteristics that function for protein specification. θ_I_ and θ_II_ stand for the coefficients among different groups predicted by type I and type II functional divergence, respectively. When these coefficients are greater than 0, it means that the site-specific selection changes the physicochemical properties of amino acids. In addition, significant differences in evolutionary rate and amino acid physicochemical properties existing between different group members were also indicated with posterior analysis [52].

### 2.5. Site-Specific Selection Assessment and Testing

The selective pressure of each residue of TF proteins was estimated with the synonymous rate (*Ks*) and non-synonymous rate (*Ka*) of nucleotide substitution. Here, *Ka*/*Ks* values were calculated with M8, M8a, M7, and M5 models. All of these models calculate the *Ka*/*Ks* value between sites with eight discrete classes and posterior distribution [53,54]. I-TASSER [55] was used to predict the tertiary structure of TF protein in Amur stickleback.

### 2.6. Recombinant N-Lobe and C-Lobe Plasmid Construction, Expression, and Purification

The cDNA fragments of N-lobe and C-lobe were amplified by the specific primers with *Nde* I and *Xba* I restriction sites (Table 1). The PCR products were digested, connected to the expression vector pCZN1, and transformed into BL21 strain. Cells were cultured until the concentration of 0.6 at OD600 in LB liquid medium adding ampicillin at 37 °C and inducted with 0.2 mM IPTG (isopropyl β-D-thiogalactopyranpside) at 15 °C overnight. The recombinant peptides were purified with His Band Resin columns (Novagen, Madison, WI, USA) after sonication. Their purity was analyzed through 12% SDS-PAGE (sodium dodecyl sulfate polyacrylamide gel electrophoresis). NanoDrop (Thermo Scientific, Wilmington, NC, USA) was used to measure the peptide concentration. It can quantify protein by detecting the absorbance value of protein at 280 nm without using protein standard.

### 2.7. Iron-Binding Assay of N-Lobe and C-Lobe of TF in Amur Stickleback

The iron-binding characteristics of N-lobe and C-lobe were evaluated with Chrome Azurol S (CAS) assay [56]. A 500 μL CAS assay solution (Sigma Aldrich, USA) was used to mix with expressed N-lobe, C-lobe, and maltose binding protein (MBP, Abcam; a protein tag, which can increase the solubility of protein and assist in protein folding but does not have any antibacterial and iron-binding capacity), and the mixture was incubated at room temperature in dark conditions for 1 h and measured the light absorbance value at 630 nm. Here, protein with different concentrations was used to detect the effect of sample concentration on iron-binding ability. The negative control is represented by an equivalent amount of elution buffer. Finally, the average of the relative absorbance of the three repetitions was calculated to represent their iron-binding activities.

### 2.8. Antibacterial Assay of N-Lobe and C-Lobe of TF in Amur Stickleback

*Escherichia coli* was first cultured in 37 °C LB medium at 200 rpm for about 6 h and diluted to the absorbance value of 1. Then, 100 μL diluted *E. coli* liquid (DH5α) and FeCl_3_ with final concentration of 1μM were added to the 96 well microtiter plate, respectively. Then, equal amounts of N-lobes, C-lobes, MBP or elution buffer (control) were added into each well to 2 μM final concentration. Finally, the light absorption value of bacterial solution at 600 nm in different time periods was measured to verify the growth of the strain. The test is repeated three times.

## 3. Results

### 3.1. Identification and Expression Patterns of TF Gene in Amur Stickleback

**Figure 1:**
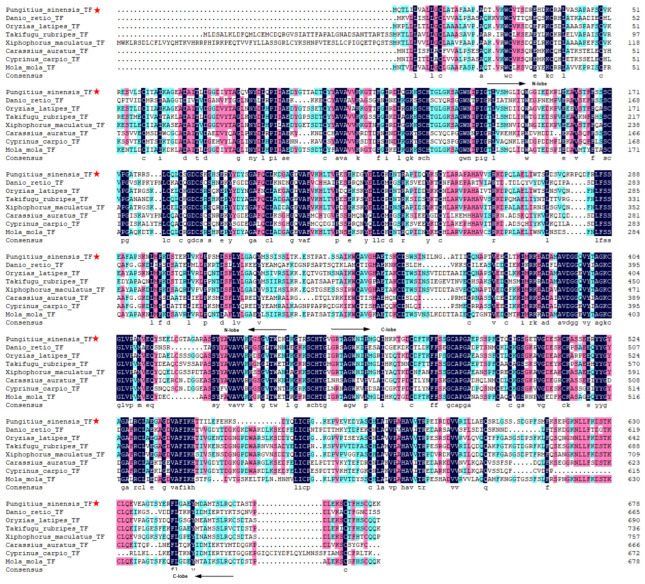
Alignment of transferrin amino acid sequences between *P. sinensis* (marked with red asterisks) and other seven fish species randomly selected. The N-lobe and C-lobe areas are marked with arrows.

A 2037-bp open reading frame was obtained from our transcriptome sequencing data of Amur stickleback [45], which encodes a TF protein with 679 amino acid residues. This *TF* transcript of Amur stickleback, similar to the zebrafish *transferrin-a* gene, has high homology with the mammalian serum transferrin. It has N-lobe and C-lobe typical functional domains, separated by a short bridge (Supplementary Data S2). This TF protein of Amur stickleback was homologous to those of other species (Figure 1).

**Figure 2:**
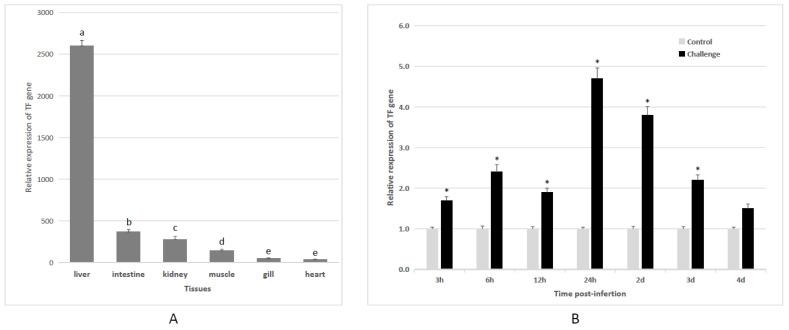
Expression patterns of *TF* gene in *P. sinensis*. (**A**) Relative expression level of the *TF* gene in six healthy tissues of *P. sinensis* (liver, intestine, kidney, muscle, gill, and heart) by qRT-PCR. Different letters indicated statistical significance at the level of *p* < 0.05. Values were described as mean ± SE (*n* = 3). (**B**) Relative expression pattern of the *P. sinensis TF* gene in the liver after *Streptococcus iniae* infection by qRT-PCR. Values were described as mean ± SE (*n* = 3). The asterisks indicated statistically significant differences from the control group (* *p* < 0.05).

Next, the tissue distribution of *PsTF* transcripts was also detected. The results indicated that the expression of *PsTF* transcripts was differentially distributed in the six examined tissues. The highest expression pattern of *PsTF* mRNA was detected in liver, followed by intestine, kidney, and muscle. Moreover, the lowest expression level of *PsTF* transcripts was detected in gill and heart (Figure 2A).

To study the effect of pathogen stimuli to the *PsTF*, the dynamic expression patterns of *PsTF* gene in liver tissue infected with *S. iniae* were analyzed (Figure 2B). The result indicated that at 24 h and 2 days after bacterial infection, the expression level of *PsTF* was the highest. Compared with the control, the increase fold of *PsTF* transcripts was about 4.7 and 3.8 at these two points of time, respectively. Therefore, the transcriptional changes of *PsTF* gene may be an immune mechanism against pathogenic stimuli.

### 3.2. Phylogenetic Analysis of the N-Lobe and C-Lobe

**Figure 3:**
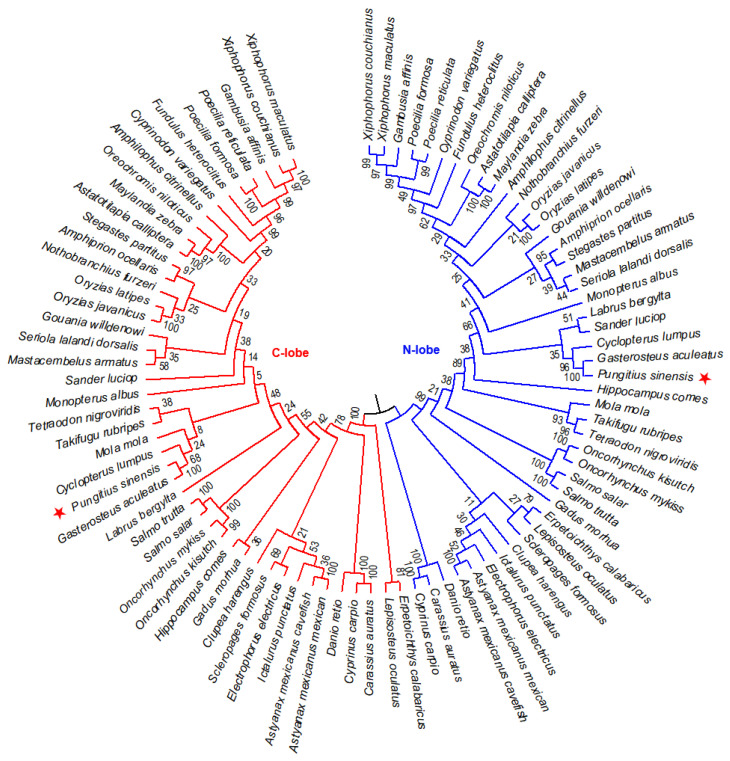
Phylogenetic relationship of N-lobe and C-lobe of TF proteins in 45 fish species. NJ tree was constructed with 1000 bootstrap replicates. Amur stickleback is marked with red asterisks.

To further elucidate the evolutionary relationship of the N-lobe and C-lobe of *TF* family genes from 45 fish species, an NJ phylogenetic tree was constructed. According to the sequence similarity and phylogeny, these members were classed into two groups, named N-lobe and C-lobe groups (Figure 3). This structure is believed to originate from a gene duplication event about 670 million years ago [18,19].

### 3.3. Motif Distribution of the N-Lobe and C-Lobe Sequences in Fish TFs

**Figure 4:**
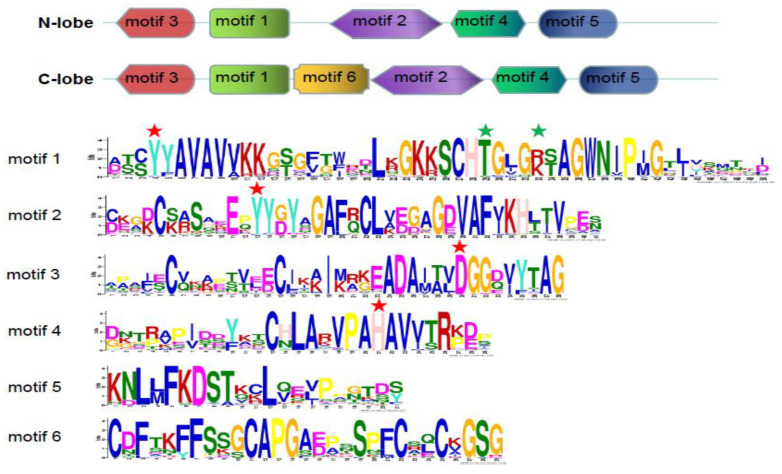
Motif distribution of N-lobe and C-lobe of TF proteins. Sequence logos of six conserved motifs are also shown here. Four iron-binding residues are marked with red stars, and two anion binding residues are marked with green stars. The size of the letter represents the degree of conservation of the amino acid. The larger the letter, the more conservative the amino acid.

To further understand the diversity of N-lobe and C-lobe in fish, six conserved motifs were identified in these sequences (Figure 4).

These N-lobes and C-lobes share most of the motif distribution, while the C-lobes specifically contain motif 6, suggesting that these sequences from ancient gene duplication have changed in evolution. The specific motif 6 in C-lobe group may confer different functions of this family protein in evolution.

### 3.4. Functional Divergence Analysis between N-Lobe and C-Lobe Groups

Type I and Type II functional divergence were estimated to investigate the residue substitution leading to adaptive functional diversification. The results showed that the type I divergence coefficient (θ_I_) of these two groups was 0.599 (Table 2), indicating the change of specific sites among them. Selective restriction on different lobe members may lead to changes in specific functions after divergence. In addition, the type II divergence coefficient (θ_II_) was also estimated with high standard error (Table 3).

To identify key residues associated with functional changes among N-lobe and C-lobe groups of TF proteins, posterior divergence probability of each site was estimated. The larger value of posterior probability indicates a great possibility of change in site-specific amino acids [52]. As a result, some key residues were identified related to functional divergence in these groups (Table 2 and Table 3).

### 3.5. Selective Pressure Estimation

**Figure 5:**
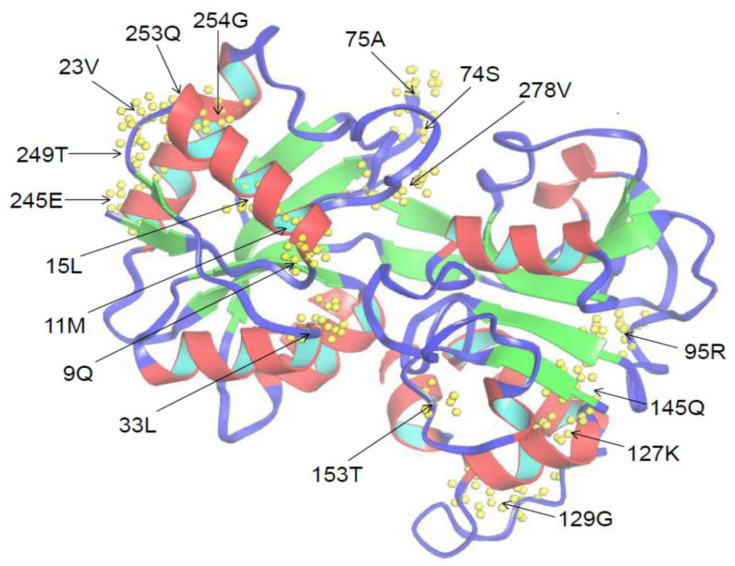
Three-dimensional structure of the N-lobe of TF protein in Amur stickleback predicted with the I-TASSER server [55]. Seventeen positive selection sites predicted by M8 model are marked with arrows.

To identify which residues experienced positive selection, the *Ka*/*Ks* value was calculated for each site of N-lobe and C-lobe sequences (Table 4). The *Ka*/*Ks* value of the C-lobe group was relatively higher than that of the N-lobe, indicating a faster change rate in C-lobe. All values of *Ka*/*Ks* were less than 1, indicating that each group had undergone purification selection. However, seventeen sites were identified to undergo positive selection in the N-lobe group predicted by M8 model (Table 4). At the same time, thirty-nine and forty-six sites were also found in the C-lobe group predicted by M8 and M5 model, respectively (Table 4). For instance, among the 17 sites of N-lobe group, 23V, 33L, 95R, 127K, and 278V sites were located in the motifs 3, 1, and 5 (Figure 5).

### 3.6. Iron-Binding Activity of the N-Lobe and C-Lobe of TF Protein in Amur Stickleback

**Figure 6:**
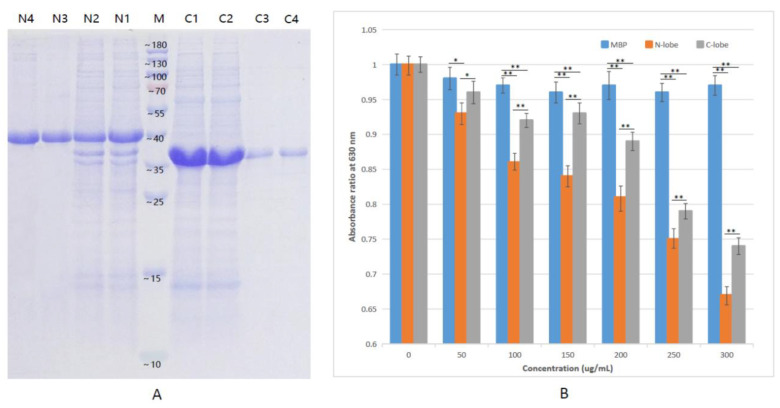
Protein expression and iron-binding activity. (**A**) SDS-PAGE analysis of Amur stickleback TF N-lobe and C-lobe. Lines: M—marker; N1 and N2—soluble fraction after sonication of N-lobe expressed cells; N3 and N4—purified N-lobe fragments after elution; C1 and C2—soluble fraction after sonication of C-lobe expressed cells; C3 and C4—purified C-lobe fragments after elution; (**B**) Iron-binding activity of the TF N-lobe and C-lobe in Amur stickleback. The x-axis represents the protein concentration, and the y-axis represents the light absorption value at 630 nm. Values were described as mean ± SE (*n* = 3). Asterisks (* *p* < 0.05; ** *p* < 0.01) indicated the statistically significant differences between treatments.

Next, N-lobes and C-lobes of TF in Amur stickleback were first expressed and purified in vitro (Figure 6A and Figure S1).

Firstly, CAS assay reagent was used to detect the potential binding ability of N-lobe and C-lobe to ferric ions (Fe^3+^). The results indicate that both N-lobe and C-lobe have the ability to bind Fe^3+^, and the level increases with the increase in concentration (Figure 6B). Compared with C-lobe, N-lobe has a stronger ability to bind iron ions. However, compared with the control, MBP has no reaction to iron-binding activity.

### 3.7. Antimicrobial Properties of the N-Lobe and C-Lobe of TF Protein in Amur Stickleback

**Figure 7:**
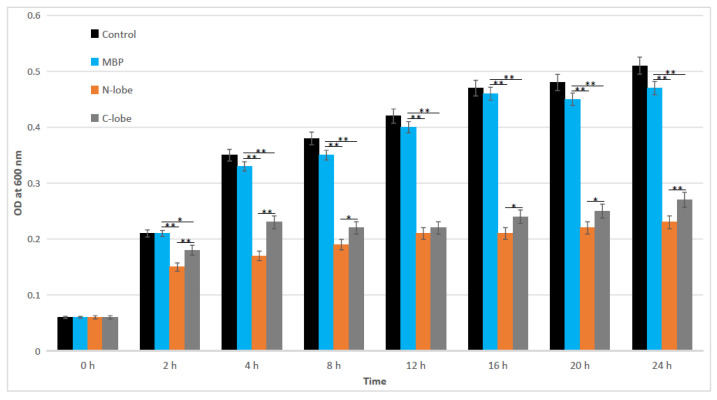
Analysis of iron-dependent bacterial growth inhibition. The x-axis represents time, and the y-axis represents the light absorption value at 600 nm. Values were described as mean ± SE (*n* = 3). Asterisks (* *p* < 0.05; ** *p* < 0.01) indicated the statistically significant differences between treatments. MBP: maltose binding protein; OD: optical density.

Next, their potential antimicrobial properties were also analyzed. The results showed that both N-lobe and C-lobe had potential antibacterial ability (Figure 7).

Compared with C-lobe, N-lobe showed stronger bacterial growth inhibitory activity. The high Fe^3+^-binding efficiency of N-lobe may play key roles in inhibiting bacterial growth. Here, the results also showed that MBP did not produce any significant changes in bacterial growth.

## 4. Discussion

Here, a transcript was obtained from Amur stickleback, which encodes a TF protein with obvious N-lobe and C-lobe domains. First, the differential tissue distribution and bacterial stimulation of this transcript were detected (Figure 2). Liver tissue is the main site for *TF* synthesis in vertebrates [57]. Therefore, high levels of *PsTF* transcripts were detected in the liver in this study. Similar results were also observed in other species of fish [10,36,37,39,40,58,59]. In some species, a large amount of *TF* gene expression was also detected in blood and other tissues [12]. This indicates that TF may play important roles in multiple tissues. Previous studies have also reported that bacterial stimulation can up-regulate the expression of *TF* gene [36,38]. In fish, high levels of *TF* transcripts were usually detected under pathological conditions [36]. In addition, insect *TF* has also been shown to be up-regulated under pathological conditions such as bacterial stimulation [60,61]. These results suggest that *PsTF* may exhibit an immune response to pathogenic infection.

Some iron- and anion-binding residues were also described by previous study [17]. In this study, four iron- and two anion-binding sites were also found in the N-lobe and C-lobe of Amur stickleback TF protein, respectively. They are residues D73, Y103, Y196, and H255 of N-lobe and D394, Y429, Y521, and H581 of C-lobe, which are located in motifs 3, 1, 2, and 4, respectively. In addition, anion-binding residues of the T128 and K132 in N-lobe and T454 and R458 in C-lobe were also found to locate in the motif 1. Mutation of some equivalent ligands resulted in a significant sharp reduction or loss of iron-binding capacity of TFs in human, rat, pufferfish, and chicken [17,62,63]. This suggests that N-lobes and C-lobes from duplication have had functional divergence in evolution.

Functional divergence identified some critical amino acid sites (Table 2 and Table 3). For instance, seventeen key functional divergent were identified between N-lobe and C-lobe groups predicted by type I functional divergence. Among them, fifteen sites were in the motifs 3 and 1. Some sites are near the Fe-binding residues and anion-binding residues described by previous study [17]. These sites may affect the Fe-binding and anion-binding function. The different evolution rates of some key residues may lead to functional differentiation of fish *TF* gene family.

In the process of evolution, selection pressure, evaluated by *Ka*/*Ks* value, usually causes specific peptides to mutate, thereby changing the structure and function of proteins. When the *Ka*/*Ks* value was greater than or less than 1, it indicated positive selection or purification selection, respectively [64]. Here, some positive selection sites were predicted in the N-lobe and C-lobe groups (Table 4). Among them, some sites are close to the iron- and anion-binding residues and may affect the ability of binding iron or anion [17]. The positive selection of fish TFs is considered to be caused by its competition with pathogenic bacterial proteins [29]. In addition, positive selection plays key roles in the diversification of TF proteins [31,35,65]. It suggests that selection pressure promotes the rapid change of TFs, which may be related to organisms’ adaptation to different environments.

Here, N-lobe was identified to have a stronger ability to bind iron ions compared with C-lobe. Previous studies have also indicated that the Fe-binding abilities are different among N-lobe and C-lobe, and N-lobe are easier to bind iron than C-lobe [16,17,66]. Moreover, the differential iron-binding activity also existed in amphioxus (*Branchiostoma belcheri*) [67], disk abalone (*Haliotis discus discus*) [68], and rock bream (*Oplegnathus fasciatus*) [10]. In addition, the linger regions of N- and C-lobes also affect the flexibility and Fe-binding rate of TF protein [69]. TF can perform some antibacterial activity through combining free iron and then creating a low iron level environment [70,71]. This study further confirmed that N-lobe are easier to bind iron than C-lobe [16,17,66]. Iron is a basic nutrient necessary for biology and participates in many important biological processes [1,2,3]. Therefore, maintaining a certain concentration of iron ions is necessary for the growth and metabolism of organisms. Under pathological conditions, the combination of TF and iron ions leads to the decrease in available iron concentration, which inhibits the growth of pathogens.

## 5. Conclusions

One *TF* gene from Amur stickleback was characterized by analyzing its expression, motif distribution, functional divergence, molecular evolution, iron-binding ability, and antibacterial properties. This *TF* gene was mainly expressed in liver and strongly induced by pathogenic bacteria. The N-lobe and C-lobe regions have different motif composition, selection pressure, and functional divergence in evolution. Moreover, different iron-binding activity and antibacterial characteristics also existed in the N-lobe and C-lobe of Amur stickleback TF protein. Therefore, in the process of evolution, the N-lobe and C-lobe domains of *TF* genes experienced different selection pressures, resulting in the changes of their motif composition and functional divergence and the differences of their iron-binding activity and antibacterial properties. This study will provide a basis for the subsequent study of *TF* family genes.

## Figures and Tables

**Table 1:** Primers used in this study.

Primers	Nucleotide Sequences (5′-3′)	Purpose
N-F	GAGAGACATATGACGGTGAAGTGGTGCGTCACGT	Protein expression
N-R	GAGAGATCTAGAAAGGGAACTGATGCTACTCATGTA	Protein expression
C-F	GAGAGACATATGTCCTCTGCCATTAAATGGTGCGCTGT	Protein expression
C-R	GAGAGATCTAGATCTGAGCGAAGTCATGGCATCCAT	Protein expression
TF-F	CTCACGCTGTTGTCAGCCGTAAG	RT-qPCR
TF-R	GCACTCTTCAACTGTAGGGGCA	RT-qPCR
actin-F	TGCTGATTGGCATGGATGAGGCC	RT-qPCR
actin-R	TCTGGAATGTCGTGAGGGACGAT	RT-qPCR

**Table 2:** Type I functional divergence between the N-lobe and the C-lobe of fish *transferrin* gene family. The critical sites are displayed by residues on the N-lobe.

Comparison	Type I θ_I_ ± SE	LRT	Posterior Probability (>0.8)	Critical Sites
N-lobe/C-lobe	0.599 ± 0.067	79.847	17	52A, 53G, 58I, 59T, 65I, 74D, 78I, 112G, 114T, 115F, 116G, 117I, 122G, 123K, 125S, 130L, 142T

**Table 3:** Type II functional divergence between the N-lobe and the C-lobe of fish *transferrin* gene family. The critical sites are displayed by residues on the N-lobe.

Comparison	Type II θ_II_ ± SE	Posterior Probability (>0.8)	Critical Sites
N-lobe/C-lobe	0.166 ± 0.219	10	52A, 53G, 59T, 64D, 72N, 73Y, 74D, 76Q, 83D, 142T

**Table 4:** Likelihood values and parameter estimates for N-lobe and C-lobe of *TF* genes. *Ka*/*Ks*: the synonymous rate (*Ks*) and non-synonymous rate (*Ka*) of nucleotide substitution; ωs: beta + w.

Gene Branches	Selection Model	*Ka*/*Ks*	Log-Likelihood	Positive-Selection Sites
N-lobe	M8 (ωs ≥ 1)	0.2902	−19,184	9Q; 11M; 15L; 23V; 33L; 74S; 75A; 95R; 127K; 129G; 145Q; 153T; 245E; 249T; 253Q; 254G; 278V
	M8a (ωs = 1)	0.2799	−19,186.2	/
	M7 (beta)	0.2739	−19,194.8	/
	M5 (gamma)	0.2858	−19,188	/
C-lobe	M8 (ωs ≥ 1)	0.3769	−21,704.9	2S; 15A; 29T; 31S; 36N; 38P; 80Q; 81D; 82L; 85R; 86A; 87D; 89T; 136V; 138N; 157S; 162S; 165V; 211Q; 220D; 224S; 228S; 238A; 243T; 246A; 252N; 266A; 270R; 281G; 282Q; 286P; 287A; 295N; 313A; 315R; 316S; 323P; 327D; 330K
	M8a (ωs = 1)	0.3187	−21,730.7	/
	M7 (beta)	0.3061	−21,757.7	/
	M5 (gamma)	03797	−21,711.6	2S; 15A; 19T; 26T; 29T; 31S; 36N; 38P; 80Q; 81D; 82L; 85R; 86A; 87D; 89T; 132Q; 136V; 138N; 157S; 162S; 165V; 211Q; 220D; 224S; 228S; 238A; 241E; 243T; 246A; 247S; 252N; 266A; 270R; 278R; 281G; 282Q; 286P; 287A; 295N; 313A; 315R; 316S; 319E; 323P; 327D; 330K

## Data Availability

The author confirms that all relevant data are included in the article and/or its Supplementary Data files.

## Supplementary Materials

The following supporting information can be downloaded at: https://www.mdpi.com/article/10.3390/ani12243458/s1, Figure S1: Quality detection and Western blot analysis of the expressed N-lobe and C-lobe segments after gel cutting; Supplementary Data S1: *Transferrin* and *Actin* sequences obtained from previous transcriptome sequencing data of Amur stickleback for primer designing in this study; Supplementary Data S2: Transferrin sequences used in this study. N-lobe and C-lobe segments are also marked.

## References

[1] Crichton R.R., Wilmet S., Legssyer R., Ward R.J. (2002). Molecular and cellular mechanisms of iron homeostasis and toxicity in mammalian cells. J. Inorg. Biochem..

[2] Neves J.V., Wilson J.M., Rodrigues P.N. (2009). Transferrin and ferritin response to bacterial infection: The role of the liver and brain in fish. Dev. Comp. Immunol..

[3] Gkouvatsos K., Papanikolaou G., Pantopoulos K. (2012). Regulation of iron transport and the role of transferrin. Biochim. Biophys. Acta.

[4] Byers B.R., Arceneaux J.E. (1998). Microbial iron transport: Iron acquisition by pathogenic microorganisms. Met. Ions Biol. Syst..

[5] Aisen P., Enns C., Wessling-Resnick M. (2001). Chemistry and biology of eukaryotic iron metabolism. Int. J. Biochem. Cell Biol..

[6] Cheng Y., Zak O., Aisen P., Harrison S.C., Walz T. (2004). Structure of the human transferrin receptor-transferrin complex. Cell.

[7] Anderson G.J., Frazer D.M. (2005). Hepatic iron metabolism. Semin. Liver Dis..

[8] Aisen P., Listowsky I. (1980). Iron transport and storage proteins. Annu. Rev. Biochem..

[9] Johnson E.E., Wessling-Resnick M. (2012). Iron metabolism and the innate immune response to infection. Microbes Infect..

[10] Perera N.C.N., Godahewa G.I., Hwang J.Y., Kwon M.G., Hwang S.D., Lee J. (2017). Molecular, structural, and functional comparison of N lobe and C lobe of the transferrin from rock bream, *Oplegnathus fasciatus*, with respect to its immune response. Fish Shellfish Immunol..

[11] Brown J.P., Hewick R.M., Hellström I., Hellström K.E., Doolittle R.F., Dreyer W.J. (1982). Human melanoma-associated antigen p97 is structurally and functionally related to transferrin. Nature.

[12] Lambert L.A. (2012). Molecular evolution of the transferrin family and associated receptors. Biochim. Biophys. Acta.

[13] Richardson D.R. (2000). The role of the membrane-bound tumour antigen, melanotransferrin (p97), in iron uptake by the human malignant melanoma cell. Eur. J. Biochem..

[14] Teng C.T. (2010). Lactoferrin: The path from protein to gene. Biometals.

[15] Giansanti F., Leboffe L., Pitari G., Ippoliti R., Antonini G. (2012). Physiological roles of ovotransferrin. Biochim. Biophys. Acta.

[16] Park I., Schaeffer E., Sidoli A., Baralle F.E., Cohen G.N., Zakin M.M. (1985). Organization of the human transferrin gene: Direct evidence that it originated by gene duplication. Proc. Natl. Acad. Sci. USA.

[17] Lambert L.A., Perri H., Halbrooks P.J., Mason A.B. (2005). Evolution of the transferrin family: Conservation of residues associated with iron and anion binding. Comp. Biochem. Physiol. B Biochem. Mol. Biol..

[18] Lambert L.A., Perri H., Meehan T.J. (2005). Evolution of duplications in the transferrin family of proteins. Comp. Biochem. Physiol. B Biochem. Mol. Biol..

[19] Jamroz R.C., Gasdaska J.R., Bradfield J.Y., Law J.H. (1993). Transferrin in a cockroach: Molecular cloning, characterization, and suppression by juvenile hormone. Proc. Natl. Acad. Sci. USA.

[20] Ellis A.E. (2001). Innate host defense mechanisms of fish against viruses and bacteria. Dev. Comp. Immunol..

[21] Skaar E.P. (2010). The battle for iron between bacterial pathogens and their vertebrate hosts. PLoS Pathog..

[22] Stafford J.L., Belosevic M. (2003). Transferrin and the innate immune response of fish: Identification of a novel mechanism of macrophage activation. Dev. Comp. Immunol..

[23] Trites M.J., Barreda D.R. (2017). Contributions of transferrin to acute inflammation in the goldfish, *C. auratus*. Dev. Comp. Immunol..

[24] Haddad G., Belosevic M. (2009). Transferrin-derived synthetic peptide induces highly conserved pro-inflammatory responses of macrophages. Mol. Immunol..

[25] Stafford J.L., Neumann N.F., Belosevic M. (2001). Products of proteolytic cleavage of transferrin induce nitric oxide response of goldfish macrophages. Dev. Comp. Immunol..

[26] Zhang X., Mosser D.M. (2008). Macrophage activation by endogenous danger signals. J. Pathol..

[27] Trinchella F., Parisi E., Scudiero R. (2008). Evolutionary analysis of the transferrin gene in Antarctic Notothenioidei: A history of adaptive evolution and functional divergence. Mar. Genom..

[28] Laurent P., Rodellar C. (2001). Characterization of a single nucleotide polymorphism in the coding sequence of the bovine transferrin gene. Mutat. Res..

[29] Ford M.J., Thornton P.J., Park L.K. (1999). Natural selection promotes divergence of transferrin among salmonid species. Mol. Ecol..

[30] Teixeira A.S., Jamieson A., Raposo J.C. (2002). Transferrin polymorphism in Central Amazon populations of pescada, *Plagioscion squamosissimus*. Genet. Mol. Res..

[31] Yang L., Gui J.F. (2004). Positive selection on multiple antique allelic lineages of transferrin in the polyploid *Carassius auratus*. Mol. Biol. Evol..

[32] Yang L., Zhou L., Gui J.F. (2004). Molecular basis of transferrin polymorphism in goldfish (*Carassius auratus*). Genetica.

[33] Rengmark A.H., Lingaas F. (2007). Genomic structure of the Nile tilapia (*Oreochromis niloticus*) transferrin gene and a haplotype associated with saltwater tolerance. Aquaculture.

[34] Antunes A., Templeton A.R., Guyomard R., Alexandrino P. (2002). The role of nuclear genes in intraspecific evolutionary inference: Genealogy of the transferrin gene in the brown trout. Mol. Biol. Evol..

[35] Andersen Ø., De Rosa M.C., Pirolli D., Tooming-Klunderud A., Petersen P.E., André C. (2011). Polymorphism, selection and tandem duplication of transferrin genes in Atlantic cod (*Gadus morhua*)-conserved synteny between fish monolobal and tetrapod bilobal transferrin loci. BMC Genet..

[36] Gao J., Ding S., Huang X., Shi X. (2013). Cloning and expression characterization of the serum transferrin gene in the Chinese black sleeper (*Bostrichthys sinensis*). Gene.

[37] Jurecka P., Irnazarow I., Westphal A.H., Forlenza M., Arts J.A., Savelkoul H.F., Wiegertjes G.F. (2009). Allelic discrimination, three-dimensional analysis and gene expression of multiple transferrin alleles of common carp (*Cyprinus carpio* L.). Fish Shellfish Immunol..

[38] Ding Z., Zhao X., Su L., Zhou F., Chen N., Wu J., Fu X., Wu F., Wang W., Liu H. (2015). The Megalobrama amblycephala transferrin and transferrin receptor genes: Molecular cloning, characterization and expression during early development and after *Aeromonas hydrophila* infection. Dev. Comp. Immunol..

[39] Denovan-Wright E.M., Ramsey N.B., McCormick C.J., Lazier C.B., Wright J.M. (1996). Nucleotide sequence of transferrin cDNAs and tissue-specific of the transferrin gene in Atlantic cod (*Gadus morhua*). Comp. Biochem. Physiol. B Biochem. Mol. Biol..

[40] Liu H., Takano T., Abernathy J., Wang S., Sha Z., Jiang Y., Terhune J., Kucuktas H., Peatman E., Liu Z. (2010). Structure and expression of transferrin gene of channel catfish, *Ictalurus punctatus*. Fish Shellfish Immunol..

[41] Tange N., Jong-Young L., Mikawa N., Hirono I., Aoki T. (1997). Cloning and characterization of transferrin cDNA and rapid detection of transferrin gene polymorphism in rainbow trout (*Oncorhynchus mykiss*). Mol. Mar. Biol. Biotechnol..

[42] Nynca J., Dietrich M.A., Adamek M., Steinhagen D., Bilińska B., Hejmej A., Ciereszko A. (2017). Purification, characterization and expression of transferrin from rainbow trout seminal plasma. Comp. Biochem. Physiol. B Biochem. Mol. Biol..

[43] Poochai W., Choowongkomon K., Srisapoome P., Unajak S., Areechon N. (2014). Characterization and expression analysis of the transferrin gene in Nile tilapia (*Oreochromis niloticus*) and its upregulation in response to Streptococcus agalactiae infection. Fish Physiol. Biochem..

[44] Livak K.J., Schmittgen T.D. (2001). Analysis of Relative Gene Expression Data Using Real-Time Quantitative PCR and the 2^−ΔΔCT^ Method. Methods.

[45] Cao J., Cheng X. (2019). Transcriptome-based identification and molecular evolution of the *Cytochrome P450* genes and expression profiling under dimethoate treatment in Amur stickleback (*Pungitius sinensis*). Animals.

[46] Yates A.D., Achuthan P., Akanni W., Allen J., Allen J., Alvarez-Jarreta J., Amode M.R., Armean I.M., Azov A.G., Bennett R. (2020). Ensembl 2020. Nucleic Acids Res..

[47] El-Gebali S., Mistry J., Bateman A., Eddy S.R., Luciani A., Potter S.C., Qureshi M., Richardson L.J., Salazar G.A., Smart A. (2019). The Pfam protein families database in 2019. Nucleic Acids Res..

[48] Edgar R.C. (2004). MUSCLE: Multiple sequence alignment with high accuracy and high throughput. Nucleic Acids Res..

[49] Tamura K., Stecher G., Peterson D., Filipski A., Kumar S. (2013). MEGA6: Molecular Evolutionary Genetics Analysis version 6.0. Mol. Biol. Evol..

[50] Bailey T.L., Williams N., Misleh C., Li W.W. (2006). MEME: Discovering and analyzing DNA and protein sequence motifs. Nucleic Acids Res..

[51] Gu X. (2001). Maximum-likelihood approach for gene family evolution under functional divergence. Mol. Biol. Evol..

[52] Gaucher E.A., Gu X., Miyamoto M.M., Benner S.A. (2002). Predicting functional divergence in protein evolution by site-specific rate shifts. Trends Biochem. Sci..

[53] Yang Z. (1994). Maximum likelihood phylogenetic estimation from DNA sequences with variable rates over sites: Approximate methods. J. Mol. Evol..

[54] Stern A., Doron-Faigenboim A., Erez E., Martz E., Bacharach E., Pupko T. (2007). Selecton 2007: Advanced models for detecting positive and purifying selection using a Bayesian inference approach. Nucleic Acids Res..

[55] Yang J., Yan R., Roy A., Xu D., Poisson J., Zhang Y. (2015). The I-TASSER Suite: Protein structure and function prediction. Nat. Methods.

[56] Schwyn B., Neilands J.B. (1987). Universal chemical assay for the detection and determination of siderophores. Anal. Biochem..

[57] Beutler E., Gelbart T., Lee P., Trevino R., Fernandez M.A., Fairbanks V.F. (2000). Molecular characterization of a case of atransferrinemia. Blood.

[58] Sahoo P.K., Mohanty B.R., Kumari J., Barat A., Sarangi N. (2009). Cloning, nucleotide sequence and phylogenetic analyses, and tissue-specific expression of the transferrin gene in *Cirrhinus mrigala* infected with *Aeromonas hydrophila*. Comp. Immunol. Microbiol. Infect. Dis..

[59] Yin X., Yang Y., Han K., Wu L., Wu H., Bian X., Wei X., Guo Z., Mu L., Ye J. (2019). Purification and functional characterization of serum transferrin from Nile tilapia (*Oreochromis niloticus*). Fish Shellfish Immunol..

[60] Guz N., Attardo G.M., Wu Y., Aksoy S. (2007). Molecular aspects of transferrin expression in the tsetse fly (*Glossina morsitans morsitans*). J. Insect Physiol..

[61] Yoshiga T., Hernandez V.P., Fallon A.M., Law J.H. (1997). Mosquito transferrin, an acute-phase protein that is up-regulated upon infection. Proc. Natl. Acad. Sci. USA.

[62] He Q.Y., Mason A.B., Woodworth R.C., Tam B.M., MacGillivray R.T., Grady J.K., Chasteen N.D. (1997). Inequivalence of the two tyrosine ligands in the N-lobe of human serum transferrin. Biochemistry.

[63] Mason A.B., Halbrooks P.J., James N.G., Connolly S.A., Larouche J.R., Smith V.C., MacGillivray R.T., Chasteen N.D. (2005). Mutational analysis of C-lobe ligands of human serum transferrin: Insights into the mechanism of iron release. Biochemistry.

[64] Hurst L.D. (2002). The *Ka*/*Ks* ratio: Diagnosing the form of sequence evolution. Trends Genet..

[65] Ford M.J. (2001). Molecular evolution of transferrin: Evidence for positive selection in salmonids. Mol. Biol. Evol..

[66] Zak O., Aisen P. (2002). A new method for obtaining human transferrin C-lobe in the native conformation: Preparation and properties. Biochemistry.

[67] Liu J., Zhang S., Li L. (2009). A transferrin-like homolog in amphioxus *Branchiostoma belcheri*: Identification, expression and functional characterization. Mol. Immunol..

[68] Herath H.M., Elvitigala D.A., Godahewa G.I., Whang I., Lee J. (2015). Molecular insights into a molluscan transferrin homolog identified from disk abalone (*Haliotis discus discus*) evidencing its detectable role in host antibacterial defense. Dev. Comp. Immunol..

[69] Wally J., Halbrooks P.J., Vonrhein C., Rould M.A., Everse S.J., Mason A.B., Buchanan S.K. (2006). The crystal structure of iron-free human serum transferrin provides insight into inter-lobe communication and receptor binding. J. Biol. Chem..

[70] Gomme P.T., McCann K.B., Bertolini J. (2005). Transferrin: Structure, function and potential therapeutic actions. Drug Discov. Today.

[71] Dietrich M.A., Zmijewski D., Karol H., Hejmej A., Bilińska B., Jurecka P., Irnazarow I., Słowińska M., Hliwa P., Ciereszko A. (2010). Isolation and characterization of transferrin from common carp (*Cyprinus carpio* L.) seminal plasma. Fish Shellfish Immunol..

