# Supplementary material for: Functional Divergence of the N-Lobe and C-Lobe of Transferrin Gene in Pungitius sinensis (Amur Stickleback)
Source: Animals (Basel). 2022 Dec 7;12(24):3458. doi: 10.3390/ani12243458 (PMC9774405; doi:10.3390/ani12243458)
Supplement: Supplementary file 1 [file animals-12-03458-s001.zip › animals-2042269-supplementary.pdf]

Supplementary Data

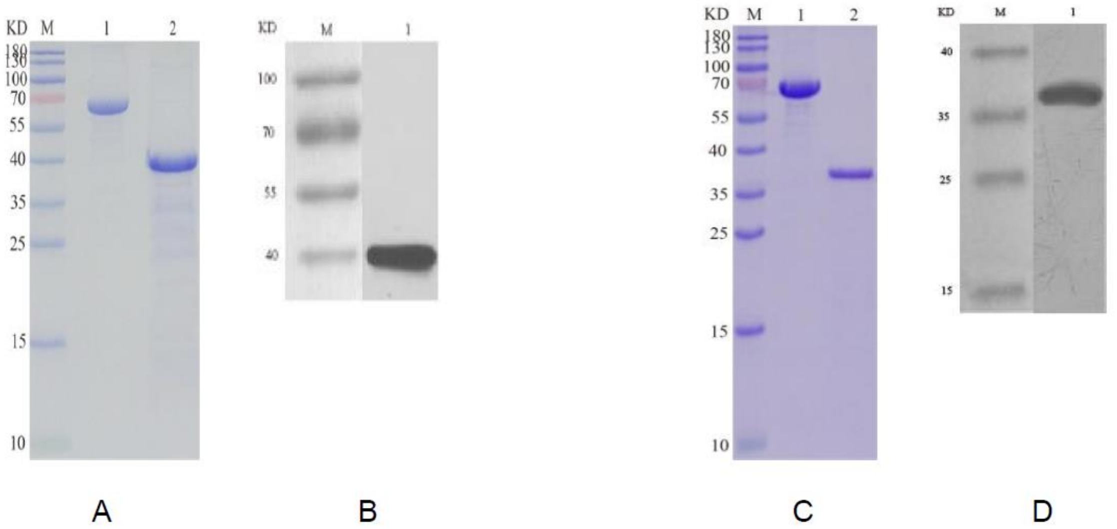

Figure S1. Quality detection and Western blot analysis of the expressed N-lobe and C-lobe segments after gel cutting. (**A**) Purification of the expressed N-lobe after gel cutting. Lines: M, marker; 1, 0.5% mg/mL BSA; 2, purified sample. (**B**) Western blot of the expressed N-lobe. Lines: M, marker; 1, purified sample. (**C**) Purification of the expressed C-lobe after gel cutting. Lines: M, marker; 1, 0.5% mg/mL BSA; 2, purified sample. (**D**) Western blot of the expressed C-lobe. Lines: M, marker; 1, purified sample.

Supplementary Data S1. *Transferrin* and *Actin* sequences obtained from previous transcriptome sequencing data of Amur stickleback for primer designing in this study.

>Transferrin
ATGCAGACTCTCATCCTCGTGGCGTTGCTCGGATGCCTCGCCACCGCGTTCGCGGCCCCCGC
TGACACGGTGAAGTGGTGCGTCACGTCGGACAGAGAACATGACAAATGTAGGGCCCTCGT
CGCAAGTGCTCCGGCTTTCTCCTGCGTGAAGAGGGAAAGCGTCCTCAGCTGCATCACTGCG
ATTAAGGCTGGTGAGGCAGATGCCATCACTCTGGATGGAGGGGATATCTACACAGCTGGAC
AGGTCAATTATGACCTGCAGCCCATTATTGCTGAGGACTACGGCACCACTGCGGACACCTG
TTACTACGCTGTTGCCGTGGTGAAGAAGGGCACTACATTTGGCATTAGAGATCTCCAGGGG
AAGAAATCCTGCCACACTGGGTTGGGGAAATCTGCGGGCTGGAACATCCCCATCGGGACC
CTTGTGTCAATGGGTTTGATTCAGTGGGGAGGCATTGAAGACAAACCCCTCGAGGAGGCGG
TGAGCACTTTCTTCTCGTCCAGCTGTGTTCCCGGGGCCACAAGGAGAAGTCTGTGTCAGCTG
TGCAGTGGAGACTGCTCCAAGAGCCACAGTGAGCGTTACTACGACTACAGCGGAGCCTTCC
AGTGTCTGAAGGATGGTGCTGGAGACGTGGCTTTTGTGAAGCACCTCACTGTCCCAGAGTC
GGAGAAGGACGGCTATGAGCTGCTGTGTAAAGATAACACCAGGGCCCCTATTGACCAATA
CAAAAGCTGCTACCTGGCCAGAGCACCCGCTCACGCTGTTGTCAGCCGTAAGGACCCACA
GCTGGCTGAACTCATCTGGACTAGCCTCGATTCAGTACAGAAACGCCCCCAGGACTTCAGG
CTCTTCTCCTCCGAAGCCTTTGCACCTTCCAAAAACCTGATGTTCAAAGACTCAACAGAAA
AGCTGGTGAAGCTGCCCCCAAGCATGGATTCCTTCTTGTATTTGGGTGCCGGCTACATGAGT

AGCATCAGTTCCCTTACGAAAGAGTCGACTCCCGCTACGTCCTCTGCCATTAAATGGTGCGC
TGTGGGTCACGCCGAGACGAGCAAGTGTGACTCGTGGAGCATCAATATTCTCAACGGTGCC
ACCATTATATGCCAGAATGCCCCTACAGTTGAAGAGTGCCTGACAAAGATCATGCGTAAAG
AGGCTGACGCAATGGCAGTTGATGGAGGACAGGTGTACACCGCTGGGAAGTGTGGTCTGG
TTCCTGCGATGGTGGAGCAGTACAGCGAAGAGCTGTGCGGCACCGCAGGAGCCAGAGCCT
CCTCTTACTATGCTGTCGCTGTGGTGAAGAAGGGCTCAGGGGTGACCTGGGACAAGCTGAA
GGGCACGAGGTCTTGCCACACAGGCGTCGGCAGAACTGCTGGCTGGAACATTCCCATGGGT
CAAATACACAAAAAGACTGGTGACTGCGACTTCACCAAGTTCTTCAGCAGTGGCTGCGCCC
CCGGAGCAGAGCCCAGCTCTCCATTCTGTACTCAGTGCATCGGCAGCGGGGAAACGGTGG
GAGATGAATCCAAGTGCAAAGCCAGCTCCGAGGAGCGGTACTACGGCTACGCTGGAGCCT
TGAGATGTCTGGCTGAGGGTGCCGGTGATGTTGCTTTCATCAAACACACAACCATTCTAGA
ATTTGAGCACAAAAGTGATTACCAGCTGATCTGCCCTGGGAAGGAGCCGGTGGAAGTCGA
AGACTATGCCTCTTGTCACCTGGCTATTGTGCCAGCCCATGCCGTGGTCACTCGTCCAGAGA
TCCGCGACGATGTTGTTCGCATTCTCCTGGCAGAGCAGTCTAGATTAGGTTCCAGCAGCGAT
GGCCCATTTAGCATGTTTAAATCAGAACCAGGAAAGAACCTTCTCTTCAAGGACTCCACCA
AGTGTCTCCAGGAAGTTGAAGCTGGAACAAGCTATGAGAGGTTTTTGGGAGCAGAGTACAT
GGATGCCATGACTTCGCTCAGACAGTGCACTGCCAGTACTCCAGATCTGGAGAAATCCTGC
ACTTTCCATTCCTGCCAGGAAAAAAAC

>*Actin* (partial)

ATGCTGATTGGCATGGATGAGGCCCCTGGGTTCCATGAGATGACGTTCCAGTCTATTAACAAGTGCGA
TATCGATGTGAGACGTGATCTTTACGGGAATATTGTTCTTTCTGGTGGGACAACCATGTTCAAGAATCT
TCCTGAGCGCCTTGGAAAGGAAATCAGCAATCTCGCACCGTCGTCTATTAAGCCGAAGGTAGTGGCTC
CGCCTGAGCGCAAGTACAGTGTGTGGATTGGCGGCTCCATCCTATCGTCCCTCACGACATTCCAGACC
ATGTGGATCAAGAAGAGCGAGTACGACGAAGCAG

Supplementary Data S2. Transferrin sequences used in this study. N-lobe and C-lobe segments are also marked.

>Pungitius sinensis

MQTLILVALLGCLATAFAAPADTVKWCVTSDREHDKCRALVASAPAFSCVKRESVLSCITAIKA
GEADAITLDGGDIYTAGQVNYDLQPIIAEDYGTTADTCYYAVAVVKKGTTFGIRDLQGKKSCH
TGLGKSAGWNIPIGTLVSMGLIQWGGIEDKPLEEAVSTFFSSSCVPGATRRSLCQLCSGDCSKSH
SERYYDYSGAFQCLKDGAGDVAFVKHLTVPESEKDGYELLCKDNTRAPIDQYKSCYLARAPAH
AVVSRKDPQLAELIWTSLDSVQKRPQDFRLFSSEAFAPSKNLMFKDSTEKLVKLPPSMDSFLYL
GAGYMSSISSLTKESTPATSSAIKWCAVGHAETSKCDSWSINILNGATIICQNAPTVEECLTKIMR
KEADAMAVDGGQVYTAGKCGLVPAMVEQYSEELCGTAGARASSYYAVAVVKKGSGVTWDK
LKGTRSCHTGVGRTAGWNIPMGQIHKKTGDCDFTKFFSSGCAPGAEPSSPFCTQCIGSGETVGD
ESKCKASSEERYYGYAGALRCLAEGAGDVAFIKHTTILEFEHKSDYQLICPGKEPVEVEDYASC
HLAIVPAHAVVTRPEIRDDVVRILLAEQSRLGSSSDGPFSMFKSEPGKNLLFKDSTKCLQEVEAG
TSYERFLGAEYMDAMTSLRQCTASTPDLEKSCTFHSCQEKN

>*Amphilophus citrinellus* (ENSACIT00000018950.1)

MKPLLLVALLGLLASASAAPQKVKWCVISNQEMRKCLDLAAAAPVFSCVEKENALDCIIAIKA
GEADAITLDGGDIYVAGLNNYDLHPIIAEDYSSASETCYYAVAVVKKGTGFNFRDLGGKKSCHT
GLGKSAGWNIPIGTLVSMNIIKWGGIEDKPLEEAVGTFFQASCVPGATRGSKLCELCKGDCSKS
QREPYYDYNGAFQCLADGVGDVAFVKHLTVPESEKSNYELLCKDNTRAPIDSYQTCYLVRVPA
HAVVTRKDPELAELISTSLQGVQNFNLFSSEAYAPAKNLMFTDSAVKLVRLPPSIDSFLYLGANY
MSIIFSLKKEPTPGITSSAIRWCAVGHAETAKCDTWSINSVTDDTTSIECQNAPTVEECLKKIMRK
EADAMAVDGGQVFTAGKCGLVPVMVEQYAQDLCSRADVTASSYFAVAVVKKGSGVTWENL
KGKKSCHTGIGRTAGWNIPMGQIYKVQNDCDFTKFFTSGCAPGADPSSPFCSLCVGSGKAVGD
EAKCKASADEQYYGYAGAFRCLVEGGGDVAFIKHTIVQENSDGNGPDWARSLRSSDYQLICPG
KAPVEITEYASCHLANVPAHAVVARPESHAEVVRILQEQQTRFGGQGTDPAFRMFQSENGKNL
LFKDSTKCLQEVPAGRSYEEFLGPEYMDSMKLLRHCSGTASDLEKSCTSHSCQQP

>*Amphiprion ocellaris* (ENSAOCT00000003602.1)

MKPLLCAALLGFLATVLAAPGPEKVKWCVKSDAELQKCLALAAAAPAFSCVKKDNTIDCIIAIK
DGNADAITLDGGDIFTAGLNNYNLQPIIAEDYGVSSDTCYYAVAVVKKGSGFNIKDLRGKKSCH
TGLGKSAGWNIPIGTLLSMNIIEWAGIEDKPVEEAVSDFFQSSCAPGAGTGSKLCELCKGDCSRS
HKEPYYDYAGAFQCLVDGVGDVAFVKHLTVPDSEKSNYELLCKDNTRAPVDNYKTCNLARVP
AHAVVTRKDAQLAELIWTSLEGVQGFNLFSSEGYASKNLMFKDSTVKLVRLPPKTDSFLYLGA
EYMSIVRSLKKEQTPGTTSSAIKWCTVSRAETAKCDTWSINTVDKVNGGNAAIECQEAPTVDEC
LIKIMRKEADAMAVDGGQVYTAGKCGLVPAMVEQYDEGLCGSSEAKASSYYAVAVVKKSSG
LTWEGLKGKKSCHTGIGRTAGWNIPMGLIYEQTKDCDFTKFFSSGCAPGADPNSPFCSQCAGSG
KAVGDESKCKASADEQYYGYAGAFRCLVQDAGDVAFIKHTTVAENSDGVSSPAWATNVKSSD
YQLICPGKAPMEITEYESCHLARVPAHAVVTRPESRSEVIRILQEQQAKFGTSVSDPSFKMFQSD
QGKNLLFKDSTKCLQEVEAGKSYEQFLGSEYTTAMSSLRHCSQTTPDLEKSCTFHSCQQA

>*Astatotilapia calliptera* (ENSACLT00000036419.1)

MKPLLLVALLGLLASAFARPGSQKVKWCVKSDQELRKCLDLAAAAPAFSCVKKENTLECIIAIK
AGEADAITVDGGDIYTAGLNNYDLHPIIAEDYGAGTDTCYYAVAVAKKNTGFGFRDLRGKKSC
HTGLGKSAGWNIPIGSLVTMNVIEWGGIEDKPLEEAVSTFFHASCAPGAARGSKLCELCKGDCS
RSQREPYYDYNGAFQCLVEGAGDVAFVKHLTVPESDKPSYELLCKDNTRAPIDNYKVCSLARV
PAHAVVTRKDPQLADLIWQSLDQVQTDHSFNLFSSEAYAPAKNLMFKDSTVRLLRVPPNTDSF
LYLGANYMSIVHSLKKEQASDDASPAIRWCAVGHAETAKCDTWSISSVSGDTTSIECQSAPTVE
ECLKKIMRKEADAVAVDGGQVFTAGKCGLVPVMVEQYVQELCSRSDVTSSSYYAVAVVKKGS
GVTWDTLKGKKSCHTGIGRTAGWNIPMGQIYKTEHDCDFTKFFSSGCAPGAEPNSPFCSLCVGS
GKAVGDQAKCKASADEKYYGYAGAFRCLVEGGGDVAFIKHTIVTENSNGNGPDWARNLRSSD
YQLICPGKGPVEISEYATCNLAQVPAHAVVTRPESHSKVVRILQEQQARFGNTGTDPSFRMFQS
ENGKNLLFKDSTKCLQEVPLGTTYEQFLGNPYMDAMKAVRHCSETTPDLEKSCTFHTCQQG

>*Astyanax mexicanus* mexican (ENSAMXT00000041616.1)
MNIILLSTILGFLGTYESCIVCRVLRMIVIQYESAMCRFCSINMNNISIYAQIFTNCVIDQDGEADAI
TLDGGDIYQAGLINYDLHPIIAEDYGQDSDTCYYAVAVVKKGTEFKFSELKGKKSCHTGLGKSA
GWNIPIGTLLSRSRIEWTGPESSPVEEAVMGYFSASCVPGAIKGSKLCQLCKGDCSRSHKEPYYD
YDGAFQCLKDGAGEVAFVKHLTVPAVDKASYELLCLDDTRKSIDSYKTCHLARVPAHAVVSR
KDPDLAARIFELLKPLQDAGLFSSEGYPAKNLIFKDSTKKLVQLPATTDSFLYLGAQYMAIIRSL
KKETSTDSASRAIKWCAVGHAETQKCDAWSINSMDETTGSVRVECQNGKTVDECLSKIMRKEA
DAMAIDGGQVYSAGRCGLVPAMVEKYTSKCCESDACPDPSYYAVAVVRADSKLTWSSLRGK
KSCHTGLGRTAGWNVPMGLLHKDTGECDFSKFFSQSCAPGADPKSKLCALCVGSGKGVGSEE
AKCKASTEELYYGYSGAFRCLVEGGGDVAFIKHTIVEEHTGGKGPAWAKSLKKSDYRLICPGH
PTESFEVSQFAKCHLAKVPAHAVVTRPESRAEVVTVLKEQQAKFGTLTPNAPFKMFESEGGKD
LLFKDSTKCLKEISDTKSYREFLGAAYLSTMDTLRQCDSSMSDLEKACTFHTCQQSV

>*Astyanax mexicanus* cavefish (ENSAMXT00005045486.1)
PMDFQQCIILACLVAAPSDPPIRWCLKSDAELRKCSDLTAKAPKLACVKRDSSEECIKAIKDGEA
DAITLDGGDIYQAGLINYDLHPIIAEDYGQDSDTCYYAVAVVKKGTEFKFSELKGKKSCHTGLG
KSAGWNIPIGTLLSRGKIEWTGPESSPVEEAVMSYFSASCVPGAPKGSKLCQLCKGDCSRSHKEP
YYDYDGAFQCLKDGAGEVAFVKHLTVPAVDKASYELLCLDDTRKSIDSYKTCHLARVPAHAV
VSRKDPDLAARIFELLKPLQVPQYMSTIRSLKKEPSTDSASRAIKWCAVGHAETQKCDAWSINS
MDDTTSSVRVECQNGKTVDECLSKIMRKEADAMAIDGGQVYSAGRCGLVPAMVEKYTPQCCG
SDACPASDPSYYAVAVVRADSKVTWSTLRGKKSCHTGLGRTAGWNVPMGLLHQDSGECDFSK
FFSQSCAPGADPKSKLCALCVGSGKGVGSEEAKCKASTEELYYGYSGAFRCLVEGGGEVAFIK
HTIVEEHTGGKGPAWAKSLKKSDYRLICPSHSTESLEISQFAKCHLAKVPAHAVVTRPESRAEV
VTVLKEQQAKFGTLTPSAPFKMFESEGGKDLLFKDSTKCLKEISDTKSYREFLGAAYLSTMDTL
RQCDSSMSDLEKACTFHTCQQSV

>*Carassius auratus* (ENSCART00000037543.1)
MNILLISFLACFVVALPSESAQKVKWCVKSQNEMKKCQHLATKSTELECYHTSSVVECMSDIW
CGCADAITVDAEDVYQAGLPNYDLRPIIAEKYKNDCCHAVAVVKRDTDFNINDLKGKTSCHSC
YQCPGGWNNPIGRLVKEHKIPWDVHDDIEKAVSQFFSSSCIPGISKAVYPNLCQACQGDCSCSN
REKYYGDEGAFQCLKSGHGQVAFMCHYGVPSSEMQNYQLLCMDGSRKSIEEYGDCYLLKEM
HHAVISRKNADSEQIYKVLKQIQDSDLFSSAAFGGKDLMFSDAATELIELPKSMDSFLYLREDYY
EAMRALRGDTQLYRKIEWCTISHAEHQNCDSLQIPRMDCRNASSVDECIKKIMRKEADAIAVDG
GQVYIAGKCGLVPVMVEQYIQERCNDGGEASSYYAVAVVRKGSGVTWKNLKGKKSCHTGLN
HSAGWIVPVLAICGQTPDCTLYNFFSKGCAPGADHQLNMCDLCKGSGKPVGDEGRCKASSEEI
YYGDDGAFRCLAEKTGEVAFIKHTIVGDYTDGKGPAWARGLKSEDFELICPELPDTTVKYTEFD
RCNLAKVPAHAVITREDARKDVVNVLKQAQVSSFSPDQLFKSEGGRNLLFSDSTKCLQEITQPL
NEFLTQKYIDMIEKTYMTDQGKPDLVKSCSYGFC

>*Clupea harengus* (ENSCHAT00000024451.1)

MHTVLLWAFLGCLASAMAAPADVKVRWCVKSDLELKKCTDLAKTAQLVCVKREGSEECIKAI
KDLQADAITLDGGDIYVAGLINYDLHPIIAEDYGTSSETCYYAVAVVKKGTDFTFKTLKGKKSC
HTGLGKSAGWNIPIGTLVAQGQIQWTGIEDKPLEDAVMEFFSASCAPGANKDSKLCKLCKDDC
SRSHKETYYDYHGAFQCLQDGAGDVAFVKHLTVPDSEKGNYELLCKDGTRKSIDDYKTCHLA
RVPAHAVVSRKDPELAKRIFDVLTPVESKLFVSDPYGGKNLMFKDSATKLVQLPSTTDSFLYLG
AEYMSIIRSLKKERGSGSASQSIKWCVTSKAERDKCDSWSVSSLDSVFCEMAGSVDECIKKIMR
KQADAMTIDGGEVYTAGKCGLVPVMVEQYVEALCKPDASGPASSYYAVAVAKKSSDITWETL
KDKKSCHTGLGRTAGWNIPMGKIHSTTGVCNFDKFFSASCAPGADKLSPLCSLCMGSGKAVGG
EDTKCQPSADELYYGYGGAFRCLVEGKGDVAFIKHTIVNEYTIEKVPAWAQGAKATDFKLIGP
GCSADIKDYEKCNLARVPAHAVVTRPEMRDDVVTILKKQQEKFGPSGSESFKLFESTDGKNLLF
KDFTQCLQDIPSGQSYKDFLGPDYMTAMTSLRQCSSSISELEQACTFHTCQQKA

>*Cyclopterus lumpus* (ENSCLMT00005033740.1)
MQTLLLVALLGCLAAVSAAPADKVKWCVTSDKELEKCAALAVKAPAFSCVKRDSVTACIAAI
KAAEADAITLDGGDIYTAGLINNNLQPIIAEDYGPSSETCYYAVAVVRKGTTFGLRDLQGKKSC
HTGLNKSAGWNIPIGTLVSMGLIQWEGIEDKPVDEAVSNYFLSSCVPGAPRNSKLCQLCRGDCS
KSHNEPYYDYSGAFQCLKDNAGDVAFVKHLTVPESEKANYELLCKDNTRAPINDYKRCHLAR
APAHAVVSRKNPQLAKLIWDSLTSVKGFNLFSSDAYAPGKNLMFKDSTTKLVQLPGNIDSFLYL
GAVYTSIIRSLKKEPTGTPSTAIKWCAVGHAETSKCDMWSINSMGDVTTTVECQTAPTVEECLK
MIMRKEADAMAVDGGQVYTAGKCGLVPAMVEQYDAAQCGIPGAQASSYYAVAVVKKSSGLT
WETLKGKRSCHTGVGRTAGWNIPMGQIYKQTGDCDFTKFFSSGCAPGSDPSSPFCSQCAGSDK
AVGDEFKCKANAAELYYGYAGALRCLVAGSGDVAFIKHSTILENADVNNDDYQLICPGKSPVP
VTDYLSCHLATVPAHAVVTRQDSRNEVVRILLAQQSAFGSTVSEGSFGLFKSDNGKNLLFKDST
QCLQQVEAGTSYDSFLGTEYMNAMNSLRMCGETTPDLEKSCTFHSCQETN

>*Cyprinodon variegatus* (ENSCVAT00000010277.1)
MNPLLLVALLGCVAAVSAAPAEKVRWCVKSDQELRKCTDLVAAAPAFSCVKKESTLECIIAIK
AGEADAITVDGGDIYTAGLNNYDLHPIIAEDYGETSDTCYYAVAVVKKGTGFGFRELGGKKSC
HTGLGKSAGWNIPIGTLITEGVLQWAGIEDKPVEEAVSTFFQSSCAPGAPRGSKLCEICKGDCSR
SQKEPYYDYSGAFQCLAEGAGDVAFVKHLTVPDSEKSKYELLCKDNTRAPIDNYKDCHLARVP
AHAVITRKDPELANLIWNSLSGVQNFNLFSSEAYAPAKNLMFKDSTQKLVRVPPNTDSFLYLGS
NYVSIVHSLKKESATGTASSGIRWCAVGHAETVKCDEWSINSVTDDSASIECQNAPTVEDCFKKI
MRKEADVMAVDGGQVYTAGKCGLVPAMVEQYDEQACTGPSDTASSYFAVAVVKKTSGVNW
ENLKGKKSCHTGVGRTAGWNMPMGQIYSQTKDCNFANFFSSGCAPGAEPSSPFCRECKGSGKA
VGDEAKCKASAEEQYYGYAGAFRCLVEGAGDVAFIKHTTVGENSDGNGPEWARGVNSNDYQ
LICPGKDPMPLSEYMSCHLAKVPAHAVVTRPEIRDNVVRILQDQQTKFGARVDSTFRMFSSTNG
KNLLFKDSTKCLQEIPSGRTFKEFLGNEYMAAMSSLRECSDTASDLEKSCTFHTCQQP

>*Cyprinus carpio* (ENSCCRT00000026706.1)

MNILLITLLACLVVALPSASAQKVKWCVKSQNEMKKCQHLETKSSELECHLKSSVTECMTSIKT
GDADAITVDGEHVYLAGLINYDLHPIIAENNKAVCSYAVAVVKSDTDFSINDLKGKTSCHSCYQ
SPGGWNIPIGRLVAQNKLPWDGPDDMPLEKAVSQFFLSSCIPGISKALYTHLCQACQGDCSCSQ
NEKYSGDEGALQCLKSGHGQVAFMCQDGIPSSERQNYQLLCMDGSRKSVEEYKDCYLAKEPH
HAVITRKDADSQHIYKVLKQILASDLFSSAAFGGKDLMFSDATTELIELPKSMDSFLYQREDYYE
AMRALKAGNPPAPRQDGKIEWCTISHAEQQKCDNLQIPSMECRRASSVEECIKKIMRKEADALA
VDGGEVYIAGKCGLVPVMVEQSNQQSCNDGGEASSYFVVAVVRKGSGVTWNTLKGRKSCHT
GLNRNAGWKVPDSAICGKTPDCTLYNFFSKGCAPGADPQSNMCELCKGSGKVVGDESKCKAS
SGEIYYGYDGAFRCLAEKLGEVAFIKHTIVGDYTDGKGPDWAKDLKSEDFELICPESPDTTVKH
TEFGKCNLAKVPAHAVITREDARKDVVNILKQAQVSSLQFCVDFPRLLKLLKEFLTKNYIDMIE
RTYETGQGEPGIQCIYDFLQYLMNSSFIAMSCCFRLTK

>*Danio retio* (ENSDART00000163194.2)
MKVLLISLLGCLVVALPSASAQKKVKWCVTTQNEQSKCRHLATKAADIECHLQPTVIDCMRSIA
AGGTDIVTVDGANVFTGGLNNYLLRPIIAEKKKECCYAVAAVKAGSGFNINELKGKSSCHSCYQ
RSGGWNTPIGKLIATNKITWEGPNEMPVERAVSEFFSSSCVPGVSKPKYPNLCKACQGDCSCSH
NEKYFGDDGAFQCLKNDNGQVAFVCHHAIPESERQNYELLCMDGSRKSVEDYKTCNFAREPA
RTVIARTDTDLQYVYDVLKQIPASDLFSSQAFGGKDLIFSDSATELMLLPKRTDSLLYLKEEYYE
AMQAFKDGNPSAPTSQTKLAMCTIGHAEKNKCDSLDHVKKSCILEASVDDCIEKIKRKEADFLA
VDGGQVYIAGKCGLVPVMAEQSNSRTASYYAVAVVRKGSGLTWNNLEGKKSCHTGLGRSAG
WKIPESAICGEKDKCTLDKFFSEGCAPGADPTSNMCKLCKGSGKPVGDESKCKPSAEEQYYGY
DGAFRCLAEKAGDVAFIKHTVVGDYTDGKGKDWAKDLKSEDFELICPNTPDTTMKYTDFEKC
NLAQVPVHAVITREDARSAVVSFLSDIQSKNNDLFTSKDGKNLLFTDGTKCLQEIKGSVDDFLT
KKYIDMIERTYKTSQNVPDLVKACTFGNCISS

>*Electrophorus electricus* (ENSEEET00000033184.1)
RIPKIKWCLKSDQELLKCTKLASEVPELGCVKQAGTKECIKAIKEGEADAITLDGGDIYTAGLKT
YDLHPIIAEEYGGESCYYAVAVVKKGSGFSFSELKGKKSCHTGLGKSAGWNIPIGTLIAHEYIKW
TGLEDSDLKKAVMEFFLASCAPGADKGSKLCQLCKGDCSRSHKEPYYDYEGAFQCLKDGVGE
VAFVKHLTVTGADKADYELLCKDGTRKSIDDYKDCHLAKVPAHAVVSRKDPELAEQIFKIVTK
LQGYGLFSSEGYSAKNLMFKDSTKSLLQLSKTIDSFTYLGAVYMATIRSLEKEISTDSSSGAIKW
CTVGHAETVKCDTWSINAVDDKGTTKIMCTRAESVDECIRKILHKEADAIAVDGGQVYTAGKC
GLVPAMVEQYDEAKCTSGGEASSYYAVAVVHKDSGLTWESLKGKKSCHTAVGRTAGWNIPM
GLIHKETEDCDFSKYFSESCAPGADPKSSLCALCAGSGKSVGGDEAKCKASSDELYYGYAGAL
RCMVEGKGEVAFVKHTTVQENADGPQWASGLKSAELKLICPGGSAEISSYTTCHLAKVPAHAI
VTRPEMRIEVVSVLKEQQTVFGVTSSNPFKMFQSEDGSNLIFKDSTKCLQDVPSTDSYKDFLGSA
YMDSMDSLRKCPDYVPELEKDCTFSEGCPFG

>*Erpetoichthys calabaricus* (ENSECRT00000008430.1)

MKLLFQCFVVFASLIYCFSGEPVPSCIKWCNKSSLEQKKCLALKAAVPGICACVQKPDTKECLQ
AIKDGNADAITLDGGDIYEAGLINYNLHPIIAEDYGENDANCYYAVAVVKKGTGFSFKELKGKK
SCHTGLGKSAGWNVPIGVLISYGLLAWNGTSEETIESAVSRFFSASCVPGADKELYPRLCQQCK
KNCVRSHDEPYYDYEGALQCLKDGKGDVAFVKHLVAQGADKDEYELLCEDGTRKPINEYKSC
FLARVPAHAVVTRKDTDLAVQIWKYLEEAGQKFPNLFKSSYYGGKNLMFKDSTVKLIKAPEN
MNSFFYLGAKYTSIIRSLTKTSDQKESRSTHSTIKWCYISHFEKNKCDAWSIASADDQGNAKIDC
VKGDSVEDCVGMIMRKEADAVTMDGGYIFSAGACGLVPVMSEVYEGSYYAVAVVRKDSGLT
WETLKGKKSCHTAFGRTAGWNIPMGIIAKDVGNCSFMDYFIKSCAPGADKNSKLCSLCRGSGL
DPENKCSASGSEMFYGYSGAFRCLAEGVGDVAFVKQTTIPENTDGRNPADWAKNLKSSDYML
LCYNGQQKTAAPSDYVNCNLAQTAAHAVMTRPESRNDVVWFLKEQQEKFGKNGSEKESFQM
FKSETKDLLFKDSTLCLSEIPPDQNYKNFLSEEYYNAILGLKKCSQTADIWKACTFHTCQQS

>*Fundulus heteroclitus* (ENSFHET00000030491.1)
MKPLLLVALFGCLAAAFAAPAEKVKWCVKSDQELRKCMDLAAAAPAFSCVKKENTLECIIAIK
AGEADAITVDGGDVYTAGLKNYDLHPIIAEDYGETSDTCYYAVAVVKKGTGFGIRELGGKKSC
HTGLGKSAGWNIPIGTLISMNIIQWAGIEDKPVEEAVSTFFQASCAPGSKLCELCRGDCSRSQREP
YYDYSGAFQCLADGAGDVAFVKHLTVPEAEKSKYELLCKDGTRQPIDNYKTCHLARVPAHAVI
TRKDPELADLIWNSLNGVQNFQLFSSDAYAPAKNLMFKDSTQKLVRVPPNTDSFLYLGADYMS
IVHSLRKEQSTGTTSTGIRWCAVGHAETEKCDTWSINSVTDDSASIECQNALTVEECFQKIMRKE
ADAMAVDGGQVFTAGKCGLAPAMVEQYQQELCGSSPATASSYYAVAVVKKSSGVTWANLKG
KKSCHTGVGRTAGWNIPMGLIYAQTKDCNFANFFSSGCAPGSEATSPFCRECKGSGKAVGDEA
KCKATAEEQYYGYAGAFRCLVEGAGDVAFIKHSIVGENSDGNGPDWARGVNSADYQLICPGK
DPVPISEFASCHLASVPAHAVVTRPEIRDRVVRVLQDQQTKFGVSAGDSTFKMFQSTSGKNLLF
KDSTKCLQEIPAGRSYEQFLGPEYMSAMASLRACSDTASDLEKSCTFHTCQQP

>*Gadus morhua* (ENSGMOT00000011457.2)
MKHLLLSVLFCCLATAFSVPVEVVRWCVTSPFEKRKCDALKLRQPVFTCVLRADATECILAIKA
GEADAITLDGGEIYTAGQHPYDLQPIISEKYGSGSSCYYAVAVVKKDTGFSFKQLRGKKSCHTGI
GKTAGWNIPIGTLLTTGQLVWSGQEDLPVEEAVSTFFSKSCVPGAGGLVGGKLCTLCPSDCSKS
ATNPYFGYAGAFKCLKDDAGDVAFINHLTVPASEKANYELLCLDGTRAPIDSYKTCNLARVPA
HAVVSRDDPELAGRIFTALTTVRGFNLFSSAGFGAANLMFKDTTQSLVRLPDGSNSFLYLGAKY
MASIQSLKKESDQTITPAIKWCAVGHAEKKKCDSWSSFSVSDGVKYVACQISLTVEGCFQRIMR
QEADAMSVDGGQVYTAGKCGLIPAMVEQYNQSLCSSAGTPQATYFAVAVVKKGSGVTWDNL
RGKRSCHTGLGRTAGWNIPMGLVHSITGSCDFGGFFPSGCAPGSEPSSTFCRQCAGSGSGVEDG
SKCSASSVEKYYGYAGAFRCLVDGAGDVAFIKHTIVADNSDGQGPAWATALKSSDYQLICPGG
VGRAEISDFASCNLAAVPSHAVVTRQDIRDDVVKMLLDQQRKFGIDGSDPLFRIYESKDGNNLL
FKDSTKCLKEIPSQTTADAFLGTGYVNAVMSLRQCPETASDLEKTCTSFSCST

>*Gambusia affinis* (ENSGAFT00000032023.1)

MKPLLLVVLLGCLAAVFAVPAAKVRWCVKSDQELRKCTDLAAAAPVFSCVRKESTLDCIIAIK
ASEADAITVDGGDVYTAGLNNYDLHPILAEDYGETSDTCYYAVAVVKKGTNFGIRDLGGKKSC
HTGLGKSAGWNIPIGTLISMNILPWAGIEDKPIEEAVSTFFQASCVPGATRGSKLCELCKGDCSRS
QREPYYDYSGAFQCLAEDAGEVAFVKHLTVPDSEKSKYELLCLDNSRAPIDSYKTCHLARVPA
HAVITRKDPELAELIRNSLDGVQNFNKFSSEAYAPAKDLMFKDSTQRLVPVPPNTDSFLYLGAD
YVSIIHSLKKEQSKGAESPSIRWCAVGHAETAKCDTWSINSVTDDTASVECQNAPTVEDCFKKI
MRKEADAMAVDGGQVYTAGKCGLVPAMVEQYQEGLCGSSPDTASSYYAVAVVKKSSGVTW
ANLAGKKSCHTGIGRTAGWNIPMGHIYAQTKDCNFSNFFSSGCAPGAEANSPFCRECKGSGKA
VGDEAKCKANAEEQYYGYAGAFRCLVEGAGDVAFIKHSIVSENSNGNGPDWARGVNADDYQ
LICPGKDPVRVQDFANCHLAVVPAHAVVTRPDVRDKVVRVLQDQQTKFGASGSDPTFRMFQS
TNGKNLLFKDSTKCLQEVSQGKNYEQFLGPEYMNAMASLRKCSETASDLEKSCTFHTCQQP

>*Gasterosteus aculeatus* (ENSGACT00000017985.1)
MQTLILAALLGCLASVFAAPTDKVKWCVTSDREHEKCRALVVKAPAFSCVKRENVLSCIIAIKA
GEADAITLDGGDIYTAGQINYNLHPIIAEDYGTTADTCYYAVAVVKKGTAFGIRDLQGKKSCHT
GLGKSAGWNIPIGTLVSMGLIQWGGIEDKPLEEAVSTYFSSSCVPGATRKSLCQLCSGDCSKTHN
ERYYDYAGAFQCLKDGAGDVAFVKHLTVPESEKDNYELLCKDNTRAPIDSYKSCYLGRAPAH
AVVSRNDPQLAELIWNSLDSVQKHPENFNLFSSEAFAPAKNLMFKDSTEKLVKLPANMDTFLY
LGAGYMSSIRSLTKETTAAASSAIKWCAVGHAETSKCDSWSINILNVATIICQNAPTVEECLTKI
MRKEADAMAVDGGQVYTAGKCGLVPAMVEQYSEEKCATAGAQASSYYAVAVVKKGSGLTW
DKLMGTRSCHTGVGRTAGWNIPMGQIHKQTGDCDFTKFFSSGCAPGAEPSSPFCTQCIGSGEAV
GDESKCKASSEERYYGYAGALKCLADDAGDVAFIKHTTILEFEHKNEYELICPGKGPMAVEDY
ASCHLAIVPAHAVVTRPEIRNDVVRILLAEQTRLGSSNTDGPFNMFKSDSGKNLLFKDATKCLQ
EVEVGTSYETFLGAEYMDAMNSLRQCTANTPDLEKSCTFHSCQENN

>*Gouania willdenowi* (ENSGWIT00000023789.1)
MTGFQFYLYSFDECKQQVKSLSCLSCYKRPSCVPDSLHSSSADSLNPPIMKMLLHVLLLLQCISV
LFAAPAENKVKWCVKSDQELRKCTDLAAAAPVFTCVKRESTLDCIVAIKAGEADAITLDGGDV
YTAGLINYDLQPIIAEDYGITSDTCYFAVAVVKKGSGFMFKDLRGKKSCHTGLGKSAGWNIPIG
TLVSMNILPWAGIEDKPVEEAVSEFFMSSCAPGATKGSKLCEACKGDCTRSQKEPYYDYNGAF
QCLVEGAGDVAFVKHLTVPDSEKSKYELLCKDNTRAPIDSYETCHLARVPAHAVVTRKDPQLA
ELIWTSLGNVQGFNLFSSDAYAPSKNLMFKDSTVKLVQVPPNTDSFLYLGAEYMSIIRSLKKEQT
PGTSPSTIKWCAVGHAETTKCDTWSINSVTGDTAAIECQNAPTVDECLKKIMRKEADAMAVDG
GQVYTAGKCGLVPAMVEQYEDELCDSSNAVTSSYYAVAVVKKGSGVTWDNLQGKRSCHTGV
GRTAGWNIPMGHIYKRTNDCDFTKFFSSGCAPGAELTSPFCSQCAGSGKAVGDEAKCKPSAEE
QYYGYAGAFRCLVEGAGDVAFIKHSIVPENSNGAGPVWARDVKSEDYELICPGKGPVPVSDYL
SCNLAKVPAHAVVTRPEARSKVIDVLQVQQANFGNRATDAPFRLFQSENGKNLLFKDSTKCLQ
QVPDGQNYQQFLGTEYMAAMSSLRHCSETTPALEKSCTFHSCQTV

>*Hippocampus comes* (ENSHCOT00000017339.1)

MKHLILSLLRPALLLSYLVAVVLSAPAENVRWCVKSDKEHEKCVAMAKKAPVFTCVKRSDSLG
CITAIHEGIADAITLDGGDIYTAGLENYKLHPIIAEDYGATSDTCYYAVAVVKKGGAFGIRDLRG
KRSCHTGLGKSAGWNIPIGTLIKLDVLKWGGIEDESIESAVGNFFSKSCAPGAEKGTSLCQACKG
DCSRSHDEPYYDYGGAFQCLVEDAGDVAFVKHLTVPDNMKADYELLCLDNTRAPIDNYKNCH
LARVPAHAVVTRKDPDLADLIWNSLNSLQGFDLFSSQAYAPAKNLLFKDSTVKLVRLPANTNSF
LYLGASYLGIVRSLNKEVIAAGSNAITWCAVSLFETRKCDKWSVNSLVDGESKILCQTAPTVEE
CMKMIMSKEADAMAVDGGEVYTAGQCGLVPAMVEQYDEAKCSQSGVSASSYYAVAVVKRG
SGVTWDNLRGKRSCHTGFGRTAGWNIPMGKIHKQTGDCDFSKFFSSGCAPGSPADSPFCSQCV
GSGKAVDDKAKCRASAEEKYYGYAGAFRCLVEGAGDVAFIKHTIVAENSDGNGAAWAANVV
SSDYELICPNKGPVPISDFESCHLAVTPAHAVVTRPESRNAVVTILQEQQSRFGKDGSDPNFRMF
ESAPEKNLLFKDSTKCLQKVPGTNDYKSFLGPDYVDAMISLRQCSDSTPDLEKLCTSHTCQQTN

>*Hucho hucho* (ENSHHUT00000087355.1)
HSQLLPSGECLDCCCVYSSVSRLYMVFIVCVRICFAAVSDFFIASCAPGATKSSKLCQLCKGDCS
RSHKEPYYDYAGAFQCLKDGAGDVAFIKHLAVPAAEKASYELLCKDGTRAPIDSYKTCHLARV
PAHAVVSRKDPDLARNIYRKLMDVKQDFKLFSSEGYAAKNLMFKDSTQKLVQLPTTIDSFLYL
GAEYMSAIRSLTKREATHAHTTSRAIKWCAVGHYEKAKCDSWSINSNAGDKILDCQVAHTVEG
CIKKIMRKEADAITVDGGEVFTAGKCGLVPAMVEQYNADLCSAPGEASSYFAVAVAKKGSGVT
WKNLKGKRSCHTGLGRTAGWNIPMGLIHKETKDCDFTKYFSKSCAPGSEKSSPFCAQCKGTGK
AVGDEAKCKASSEEQYYGYTGAFRCLVEGAGDVAFIKHTIVPESTDGSGPVWAKDLKSSDFEL
LCQDGTTQPVSKFLECHLAKVPAHAVITRPETRKEVVSILLEQQVGVKTYSQICFYLRQIISQFSQ
G

>*Ictalurus punctatus* (ENSIPUT00000022799.1)
MKLSVIGVVLGCVALAKAAPMDPRIRWCLKSEQEAQKCRQLASKSDLLSCVKLEGSMECIKAI
QNGEADAITLDGGDIYTAGLAPYNLHPIIAEHYGTDEETCYYAVAVAKKGTNFGFHDLRGKKT
CHTGLGQTAGWNIPIGTLVEKGHIKWAGIEDKPVEEAVKEFFPASCVPGAINSLRLCELCKGDCS
RSHKEPYYGHDGAFQCLKDGAGDVAFVNHFTVLTADKANYELLCKNGSRKSVDEFKTCNLAQ
VPADAVVSRKDEDLAKRIFEVLDKLKDKGLFTSEGFSAKNLMFKDSTKGLLKLPESTDSFLYLG
EEYVAISHALTKGFRTGSKSHEITWCTVGHAEKNKCDMWTFMSVDDQTNIRIECQDGSTVDNCI
SKIMRKEADAMAVDGGQVYSAGKCGLVVAMVEEYNNESKCNFGSADASSYYAVAVVRKDSD
VTWETLIGRKSCHTGLGRTAGWNIPMGLLHDHTKECNFSKYFSESCAPGADPKSNLCKLCKGA
EGIRGKLDKCKASTAERYYGYAGAFRCLAEGAGDVAFVKHSTVAENTDGNGPEWAKAFKSAD
FKLICPGGSAEITEYEKCYLAKVPAHAVVTHPEKRDDVVSVLKEQQAVYGHSSSWVVFNMFKS
EGKQNLLFKDSTKCLQEVPPGKNYKEFLGKEYIRTIDSLRECASSKTELEKACTIHSCQKKT

>*Labrus bergylta* (ENSLBET00000025664.1)
MKALLLVLLGCLATVFAAPLEKVKWCVKSDQEFQKCLSLEAKTSAFTCVKRDSTLNCIIAIRAG
EADAITLDGGDIYTAGLNNYNLQPIIAEDYGTSSETCYYAVAVVKKGSGFSFSQLQGKKSCHTG
LGKSAGWNIPIGTLVSMNLIHWEGIEDKPIEEAVSEYFTASCAPGAVPGSNLCKQCKHDCSRTHT

EPYYDYSGAFQCLAEDAGDVAFVKHLTVPDSEKSKYELLCKDDTRAPIDSYQTCHLAKVPAHA
VVTRKDQQLADLIWESLSSVQPADLFSSEAYAPSKNLMFKDSTVKLVRVPPNTDSFLYLGAEY
MSIVRSLKKEQTAGTSSSAIRWCGVGQVETSKCDTWSINSVVDDGSTLVECVRGNTVDECLSKI
MRKEADAMAVDGGQVYTAGKCGLVPVMAEQYDEGLCSTIGATASYYAVAVVKKNSGVTWE
TLKNKKSCHTGVGRTAGWNIPMGHIHKLTQDCDFTKFFSSGCAPGSEPSSPFCSQCAGSGKAVG
DESKCKASAEEQYYGYAGAFRCLVEGAGDVAFIKHTTVPENSNGNGPAWASSSRAEDFELICP
GKAPVPITEFQSCHLAKVPAHAVVTRPESRDHVVTTLKDQQGKFGNSAGEFTFKLFQSDNGKN
LLFKDSTKCLQEIQAGTSFGAFLGTEYMDAMNSLRVCGQSTPDLERSCTFHSCQQKN

>*Lepisosteus oculatus* (ENSLOCT00000011952.1)
MKLVLCSLVLGCLASALAAPQSQPVRWCLKSAAEKKKCDALVKASPRFSCVQKSSTLDCIKAI
KEAHADAITLDGGDVYEAGLKNYNLHPVVAEDHGPGSDTCYYAVAVVKKGSGFSFSDLKGKK
SCHTGLGKSAGWNIPIGTLVSMGLTDWKGAEDEPIEKAVSRFFSGSCVPGADKSLYPGLCSQCK
GDCSRSQKEPYYDYSGAFQCLKDGKGDVAFVKHLTVPDSEKPNYELLCKDGTRKAISEYASCH
LAQVPAHAVVSRKSPALAKRIWESLEDAKSKFSLFRSDAYGGKDLVFKDSTQKLVHLPETMDS
FLYLGAEYMSVIRSLKGETIGKSRSGAVQWCTTGHFEMNKCDDWAINSVAAKSRTRVECAREA
TVEDCVKKIMRGDADAISLDGGEIYSAGKCGLIPAMVEQYDADQCKVSGATGSYFAVAVVKK
GSGLTWKTLKGKKSCHTGIGRTAGWNVPMGLIHKEIRSCNFSDYFSQSCAPGAKKGSSLCALC
VGSGKKVGETNKCVASSEENYYGYTGAFRCLVEGGGDVAFVKHTTVEENTAGRNPADWAKN
LKASDFELLCPTGPPVAAVNQYKTCNLAQVPSHGVMVRPESRSKVIAFLNEQQELFGRGKKGN
FTLFTSTEGKNLLFKDTTQCLQEVPLGQTSKEFLKTDYYDAVTKLSECETSVAQLVKSCTFHTC
QKH

>*Mastacembelus armatus* (ENSMAMT00000019068.2)
MKLLLLVALIGGLATMFAAPAEKVKWCVKSEPEYRKCLDLAAKAPAFSCVRRDNTIDCIIAIKA
SEADAITLDGGDIYTAGLNNYDLHPIIAEDYGPNYSDTCYLAVAVAKKGTLFGFKDLRGKKSCH
TGLGKSAGWNIPIGTLVSMGIIQWGGIEDKPVEEAVSKFFQASCAPGATRGSKLCELCKGDCSRS
HAEPYYDYDGAFQCLVEGAGDVAFVKHLTVPEAEWSKYELLCKDNTRAPMSMYESCNLARVP
AHAVVTRKDSQLAELIWTSLNSVQGFNLFSSEGYGPSKNLIFKDSTQRLVQLPPNTDSFLYLGAE
YMSVIRSLKKEQATATASTAIKWCAVGHAETAKCDTWSISVGDDSASIECQNAPTVEECLKKIM
RKEADAVAVDGGQVYTAGKCGLVPAMVEQYDSDQCSISNAKSSKYLAVAVVKKGSGVTWDT
LKGKRSCHTGIGRTAGWNIPMGHIHNLTNDCDFTKFFSSGCAPGADPSSPFCSQCIGSGKAVGD
ESKCKASADEQYYGYAGAFRCLVDGAGDVAFVKHTIVQENSDGNGPEWAKGVNSADYELICP
GKGPVPVTDFASCNLAEVPAHAVMTRPENRGEVVRILQEQQAKFGPTGSDASFRLFQSEQGKN
LLFKDSTKCLQEIPVGTNYEKFLQHQYILAMNSLRQCSETAPDLEKSCTFHNCQPKN

>*Maylandia zebra* (ENSMZET00005035241.1)
MKPLLLVALLGLLASAFARPGSQKVKWCVKSDQELRKCLDLAAAAPAFSCVKKENTLECIIAIK
AGEADAITVDGGDIYTAGLNNYDLHPIIAEDYGAGTDTCYYAVAVAKKNTGFGFRDLRGKKSC
HTGLGKSAGWNIPIGSLVTMNVIEWGGIEDKPLEEAVSTFFHASCAPGAARGSKLCELCKGDCS

RSQREPYYDYNGAFQCLVEGAGDVAFVKHLTVPESDKPSYELLCKDNTRAPIDNYKACSLARV
PAHAVVTRKDPQLADLIWQSLDQVQTDHSFNLFSSEAYAPAKNLMFKDSTVRLLRVPPNTDSF
LYLGANYMSIVHSLKKEQASDDASPAIRWCAVGHAETAKCDTWSISSVSGDTTSIECQSAPTVE
ECLKKIMRKEADAVAVDGGQVFTAGKCGLVPVMVEQYVQELCSRSDVTSSSYYAVAVVKKGS
GVTWDTLKGKKSCHTGIGRTAGWNIPMGQIYKTEHDCDFTKFFSSGCAPGAEPNSPFCSLCVGS
GKAVGDQAKCKASADEKYYGYAGAFRCLVEGGGDVAFIKHTIVTENSNGNGPDWARNLRSSD
YQLICPGKGPVEISEYATCNLAQVPAHAVVTRPESHSKVVRILQEQQARFGNTGTDPSFRMFQS
ENGKNLLFKDSTKCLQEVPLGTTYEQFLGNPYMDAMKAVRHCSETTPDLEKSCTFHTCQQG

>*Mola mola* (ENSMMOT00000025312.1)
MNTVLLVALLGCLGAAFASPAQTVRWCLKSVQEYEKCRRLAVVEPRISCFRKENTLDCIIAIKD
KLADAITLDGGDIYTAGLINYDLHPIIAEDYGTSSDTCYFAVAVVKRGTGFGIKELKGKKSCHTG
LGKSAGWNIPIGTLLSMQLIDWAGTEDSPIEDAVMTYFTASCAPGAQKDTKLCQLCRGDCSRSH
KEPYYDYAGAFQCLADGAGEVAFVKHLTVPNSEKSKYELLCKDNTRQPIDNYKECHLARVPA
HAVCSRKDPRLAELIWNVLNSDSFNLFSSQAYAPAKNLMFKDSAVRLVRLPQNTDAFLYLGAE
YLSIIRSLKREPATSATSTAIKWCAVGKAETDKCDTWSVNSLKDDTTAIECQTGNTVEDCMTKI
MRKKADAMAVDGGEVYTAGKCGLVPVMVEQYDECAASSYFAVAVVKKGRGITWETLRGKR
SCHTGIGRTAGWNVPMGLIHKQTGDCDFTKFFSSGCAPGANASSPFCAQCVGSRNPVGEDFKC
KASAGEQYYGYTGALRCLIEDAGDVAFIKHSTFGDVTGSKELTPLNHNHVLICPGKLPVPVTDY
SSCHLALVPAHAVVTRPESHADVVRVVLAQQAMFKNGGTGSSFSLFRSPNGKNLLFKDSTKCL
QEIPAGTSFEQFLGDEYMTAIKSLRQCTDSTPALEKSCSFHSCQQKH

>*Monopterus albus* (ENSMALT00000026314.1)
MKPLLLVALLGYLATVFAADQVKWCVTSEAELRKCTDLSAKAPVFSCVRKENNLACIVAIKAG
EADAITLDGGDLYIAGLINYDLHPIIAEHYGPSSEACYYAVAVVKKGTGFGINELKGKKSCHTGI
WKSAGWVLPVGTLVSMDIIQWKGIEDKPVEEAVSDFFVASCVPGATRGSKLCQLCKGDCSKSP
NEPYNDYEGAFLCLKEDAGQVAFVKHLTVPDAEKNGYELLCLDNTRAPIDSYKTCHLARVPAH
AVVTRKDPQLAELIWNSLSSVQGFSLFSSESYGVKNLLFKDSTQNLVRLPPNTDSFLYLGAQYM
SVMRSIKKELTGSTSPNTIEWCNVGHAETAKCDAWSVNSVDEEGNAAIECRSAPTVEECLKKIM
HKEADAMAIDGGQVYTAGKCGLVPVMMEQYEAGGCSNSNVRTASYYAVAVVKKESGLTWE
TLKGKKSCHTGVGRTAGWNIPMGFIHKQTNDCDFTKFFSSGCAPGSDPASPFCRQCIGSGKAVG
DESKCKANTDEQYYGYAGAFRCLVDGAGDVAFVKHSTPGENSDGKGPDWAQNVKSSDYVLIC
PGKDPVPVSDFVSCNLAVVPTHAVVTRPETRNKVVNVLLKQQALFGSGASDSSFKMFQSEGGK
NLLFKDSTLCLQEVPPGTSYDKFLEEGYMTAMRSLRQCSETAPDLEKICTSHTCQQQD

>*Nothobranchius furzeri* (ENSNFUT00015028535.1)
MQGPGPVGSSIKASAPSQTVCSAAQIRRPTHTHTQTMKPLLLVALFGCLATVFAAPAETVKWC
VISEPELQKCTELATAASIFSCVKRESPLDCIIAIKAGEADAITVDGGDVYTAGLNNYDLHPIIAED
YGAASETCYYAVAVVKKGTGFGIRDLRGKKSCHTGLGKSAGWNMPIGTLVAQDIIQWAGIEDR
KVEEAVSSFFNASCVPGAERSSGLCKLCKGDCSKSQREPYYDYNGAFQCLADGVGDVAFVKHL

TVPDSDKANYELLCKDNTRQSIDNYRLCNLARVPAHAVISRKDPQLTQLIWNSLSGVQNFNLFS
SKPPYKNLMFKDSTQKLVKLPPNMDSFLYLGAQYMSILRSLKKEQTPGVASPAIKWCAVGHAE
TAKCDTWSINSLTGNSASIECQNAPTVDECLKKIMRKEADAMAVDGGQVYTAGKCGLVPAMV
EQYDSALCGSSGAAASSYYAVAVVKKSSGVTWANLKGKRSCHTGFGRTAGWNIPMGLIYDQT
KDCDFTKFFSSGCAPGLEPSSPLCRQCKGSGQTVGDEAKCKASANEQYYGYTGALRCLAEDAG
DVAFVKHTTVLESSDADSYRLICPGKDPVPVSEYQTCHLASVPAHAVVTRPESRSEVVRILHDQ
QTKFGTSGSDPTFRLFQSENGNNLLFKDSTKCLQEVASGTSYSQFLGQEYMSAMSGLRQCTETA
SDLEKSCSFHSCQQD

>*Oncorhynchus kisutch* (ENSOKIT00005094898.1)
MKLLLVSALLGCFATVYAAPAEGMVRWCVKSEKELKKCHDLAANVAGFSCVRRDDSLECIQA
IKREEADAITLDGGDIYIAGLHNYNLQPIIAEDYGEDSDTCYYAVAVAKKGTEFGFLDLRGKKSC
HTGLGKSAGWNIPIGTLVTVGQIQWAGIEDRPVESAVSDFFNASCAPGANRDSQLCQLCMGDCS
RSHNEPYYDYSGAFQCLKDGAGEVAFIKHLTVPAAEKASYELLCKDNTRAPIDSYKTCHLARVP
AHAVVSRKDPRLANLIYSKLMAVTNFNLFSSDGYAAKNLMFKDSTQNLVQLPMTTDSFLYLGA
EYMSTIRSLTKAQATGVTSRAIKWCAVGHKEKVKCDAWTINSFTDGDSRIECQDAPTVDECIKK
IMRKEADAIAVDGGEVFTAGKCGLVPVMVEQYDEVRCSAPGEASSYFAVAVAKRGSGLTWTT
LKGKRSCHTGLGRTAGWNIPMGLIHRRTMNCDFTTYFSKGCAPGFEVDSPFCAQCKGSGQSVG
GDGSKCKASSEEQYYGYNGAFRCLVEDAGDVAFIKHTIVPEMTDGSGPVWAQNLMSSDFELLC
QDGTTKPVTHFRECHLAKVPAHAVITRPESRGEVVSILLEQQARFGSSGSDSSFNMFKPDFGKNL
LFKDSTKCLQEIPSGTKFQGFLGEEYMIAMQSLRECSNSTSDLEKACTFHSCQQKK

>*Oncorhynchus mykiss* (ENSOMYT00000115310.1)
MKLLLVSALLGCLATVYAAPAEGMVRWCVKSDKELQKCHDLAANVAQFSCVRRDNSLECIQA
IKREEADAITLDGGDIYIAGLHNYNLQPIIAEDYGEDSDTCYYAVAVAKKGTDFGFLNLRGKKS
CHTGLGKSAGWNIPIGTLVTVGQIQWAGIEDRPVESAVSDFFNASCAPGANKDSKLCQLCKVDC
SRSHNEPYYDYAGAFQCLKDGAGEVAFIKHLTVPAAEKASYELLCKDNTRAPIDSYKTCHLSRV
PAHAVVSRKNPELANRIYSKLMAVENFNLFSSDGYAAKNLMFKDSTQNLVQLPMTTDSFLYLG
AEYMSTIRSLTKAQATGATSRAIKWCAVGHNEKVKCDAWTINSFTDGDSRIECQDAPTVDECIK
KIMRKEADAIAVDGGEVFTAGKCGLVPVMVEQYDAVQCSAPGEASSYFAVAVAKKGSGVTW
NTLQGKRSCHTGLGRTAGWNIPMGLIHKETNNCDFTTYFSKGCAPGFEVDSPFCAQCKGSGQS
VGGDRARCKASSEEQYYGYTGAFRCLVEGAGDVAFIKHTIVPENTDGSGPVWAQDLKSSDFEL
LCHDGTTQPVTKFRDCHLAKVPAHAVITRPESRGEVVSILLEQQARFGSSGSDSSFNMFQSDLG
KNSLFKDSTKCLQEIPSGTKFQDFLGEEYMIAMQSLRECSNSTSDLEKACTFHSCQQKE

>*Oreochromis niloticus* (ENSONIT00000054363.1)
MKPLLLVTLLGLLASAFAAPGSQKVKWCVKSDQELRKCSDLAAAAPAFSCVKKENTLECIVAI
KAGEADAITVDGGDIYTAGLNNYDLHPIIAEDYGTGTDTCYYAVVVAKKNTGFGFRDLRGKKS
CHTGLGKSAGWNIPIGSLVSMDVIEWGGIEDKPLEEAVSTFFHASCAPGATRGSKLCELCKGDC

SRSQREPYYDYNGAFQCLVEGAGDVAFVKHLTVPESDKPMYELLCKDNTRAPIDNYKACSLAR
VPAHAVVTRKDPQLADLIWQSLDRVQTDHSFNLFSSEAYAPTKNLMFKDSTVKLVRVPPNTDS
FLYLGANYMSIVHSLKKEQASDVASPAIRWCAVGHAETAKCDTWSINSVSDDTASIECQSAPTV
EDCLKKIMRKEADAVAVDGGQVFTAGKCGLVPVMVEQYDQEMCGNSNAPASSYYAVAVVK
KGSGVTWENLKGKKSCHTGIGRTAGWNIPMGLIYNREHDCDFTKFFSSGCAPGAEPTSPFCSLC
VGSGKAVGDEAKCKASADEKYYGYAGAFRCLVEGGGDVAFVKHTIVTENSDGNGPDWARNL
RSSDYQLICPGKGPVEISDYATCHLAVVPAHAVVTRPDNHRKVVRILQEQQARFGNTGTDPSFR
MFQSENGKNLLFKDSTKCLQEVAEGSTYEQFLGEAYMDAMKALRICSETASDLEKSCAFHTCQ
QG

>*Oryzias javanicus* (ENSOJAT00000037182.1)
MKPLLLLTLLGCLAAALAAPPQKVKWCVKSDQEFRKCTDLAAAFPAFSCVKKESTLDCIIAIKA
GEADAITVDGGDVYTAGLNNYDLQPIIAEDYGASSETCYYAVAVVKKGTAFGIRDLRGKKSCH
TGLGKSAGWNIPIGTLVSMGIIQWAGIEDKPVEEEVSTFFQASCVPGATRGSKLCEICKGDCSRS
QKEPYYDYNGAFNCLAEGAGEVAFVKHLTVPDQEKSKYELLCRDNTRAPIDDYKKCNLARVP
AHAIVTRKDPQLAELIWTSVNSLQSSQTFNLFSSDAYAPARNLMFKDSTQRLVKLPSNTDSFLYL
GAQYMSTIRSLKKEQSPGTASNAIKWCAVGHAETAKCDTWSINSVTDDTAAIECQNAPTVDEC
LKKIMRKEADAMAVDGGEVYTAGKCGLVPVMVEQYDAEQCSSSEGQASSYYAVAVVKKDSG
VTWENLQGKRSCHTGMGRTAGWNIPMGRIYEQTKDCDFTKFFSSGCAPGAEPTSPFCTNCKGS
GKAVGDEAKCKARADEQYYGYAGAFRCLAEGAGDVAFIKHTIVSENTDGKGPEWARSLKSDD
YQLICPGKAPMAISEFANCNLAVVPAHAVVTRPESRSDVVRILQVQQTFFGASGSDPSFKLFQSE
NGNNLLFKDSTKCLQEVSAGKTFDQFLGAEYMEAMNSLRQCSDSTSDLEKSCTFHSCQQA

>*Oryzias latipes* (ENSORLT00020019142.1)
MKPLLLLTLLGCLAAALAVPAQKVKWCVKSDQEFRKCSDLAAASPAFSCVKKESTLDCIIAIKA
GEADAITVDGGDVYTAGLNNYDLHPIIAEDYGTSSETCYYAVAVAKKGTTFGIRDLRGKKSCH
TGLGKSAGWNIPIGTLVSMDIIQWAGVEDKPVEEEVSTFFQASCVPGATRGSKLCELCKGDCSR
SQKEPYYDYNGAFNCLAEGAGDVAFVKHLTVPDQEKSKYELLCRDNTRAPIDDYKKCNLARV
PAHAIVTRKDPQLAELIWTSLNSVQNFNLFSSEAYAPSKNLMFKDSTQRLVRLPQNTDSFLYLG
AQYMSIVRSLKKEQTVGTNSNAIKWCAVGHAETAKCDTWSINSVTDDTAAIECQNAPSVEECL
KKIMRKEADAMAVDGGEVYTAGKCGLVPAMVEQYDAELCSSSGGQASSYYAVAVVKKDSGV
TWENLKGKKSCHTGIGRTAGWNIPMGRIYDQTKDCDFTKFFPSGCAPGAEASSSFCTLCKGSGK
AVGDEAKCKARPEEQYYGYAGAFRCLAEGAGDVAFIKHTIVGENTDGNGPDWARSLKSDDYQ
LICPGKGPVPISEYASCNLAVVPAHAVVTRPESRSDVVRVLQVQQTFFGASGSDPSFKLFQSQNG
NNLLFKDSTKCLQEVPAGTSYDQFLGSGYMEAMTSLRKCSDTASDLEKSCTFHSCQQT

>*Poecilia formosa* (ENSPFOT00000009400.2)
MKPLLLVVLLGCLAAVLAAPAAKVRWCVKSDQELRKCTDLAAASEAFSCVRKENTLECIIAIK
ASEADAITVDGGDVYTAGLNNYDLHPIIAEDYGETSDTCYYAVAVVKKGTGFGIRDLGGKKSC

HTGLGKSAGWNIPIGTLISQNILQWAGIEDKPIEEAAVSTFFQASCAPGATRGSKLCELCKGDCS
RSQREPYYDYSGAFQCLVEDAGEVAFVKHLTVPDSEKSNYELLCLDNTRATIDSYKTCHLARVP
AHAVITRKDPELAKLIWESLSGVQNFNLFSSEAYGPAKDLMFKDSTQKLVPVPPNTDSFLYLGA
DYVSIIHSLRKEQSTGPESTSIRWCAVGHAETAKCDMWSINSVTEDTASVECQNAPTVEDCFKKI
MRKEADAMAVDGGQVYTAGKCGLVPAMVEQYQQELCGSSPDTASSYYAVAVVKKSSGVTW
ANLQGKKSCHTGIGRTAGWNIPMGHIYAQTKNCQFDTFFSSGCAPGAEANSPFCRECKGSGKA
VGDEAKCKASAEEQYYGYAGAFRCLVEGAGDVAFIKHSIVSENSDGNGPDWARGVNSADYQL
ICPGKDPVPVEDFVSCHLAAVPAHAVVTRPDVRDKVVRILQDQQTKFGTDGSDSTFRMFQSTN
GKNLLFKDSTKCLQEVTSGKTYDQFLGQEYMNAMSSLRQCADTASDLEKSCTFHACQQP

>*Poecilia reticulata* (ENSPRET00000004349.1)
MWKLRSGLCFPVYQHTKVHRRPHIRKPEQTVVFYLLASAGRLCYKSHGPVTESLLCPTGQXTP
QSTSMKSLLLVVLLGCLAAVLAAPAXKVRWCVKSDQELRKCTDLAAASEAFSCVKKESTLECII
AIKASEADAITVDGGDVYTAGLNNYDLHPIIAEDYGETSDTCYYAVAVVKKGTGFGIRDLGGK
KSCHTGLGKSAGWNIPIGTLISQNIIQWAGIEDKPIEEAVSTFFQASCAPGATRGSKLCELCKGDC
SRSQREPYYDYSGAFQCLVEDAGEVAFVKHLTVPDSEKSNYELLCLDNTRAPIDNYKACHLAR
VPAHAVITRKDPELAKLIWESLRGVQNFNLFSSEAYAPAKDLMFKDSTQKLVPVPPNTDSFLYL
GADYVSIIHSLRKEQSAGPESSSIRWCAVGHAETAKCDTWSINSVTEDTASVECQNAPTVEDCF
KKIMRKEADAMAVDGGQVYTAGKCGLVPAMVEQYQQELCGSSPDTASSYYAVAVVKKSSGV
TWANLQGKKSCHTGIGRTAGWNIPMGHIYAQTNNCQFDTFFSSGCAPGAEANSPFCRECKGSG
KAVGDEAKCKASAEEQYYGYAGAFRCLVEGAGDVAFIKHSIVSENSDGNGPDWARGVNSADY
QLICPGKDPVPVEDFVSCHLAAVPAHAVVTRPDIREKVVGILQDQQTKFGASGSDSTFRMFQST
NGKNLLFKDSTKCLQEVTAGKNYEQFLGQEYMNAMSSLRQCADTASDLEKSCTFHTCQQP

>*Salmo salar* (ENSSSAT00000106960.1)
MKLLLVSALLGCLATAYAAPAEGIVRWCVKSEQELRKCHDLAAKVAQFSCVRKDGSFECIQAV
KGGEADAITLDGGDIYTAGLTNYGLQPIIAEDYGEDSDTCYYAVAVAKKGTAFGFKTLRGKKS
CHTGLGKSAGWNIPIGTLVTESQIQWAGIEDRPVESAVSDFFNASCAPGATMGSKLCQLCKGDC
SRSHKEPYYDYAGAFQCLKDGAGDVAFIKPLAVPAAEKASYELLCKDGTRASIDSYKTCHLAR
VPAHAVVSRKDPELANRIYNKLVAVKDFNLFSSDGYAAKNLMFKDSAQKLVQLPTTTDSFLYL
GAEYMSTIRSLKKSQATGASSRAIKWCAVGHAEKGKCDTWTINSFADGESKIDCQDAPTVEECI
KKIMRKEADAIAVDGGEVYTAGKCGLVPVMVEQYDADLCSAPGEASSYYAVAVAKKGSGLT
WKTLKGKRSCHTGLGRTAGWNIPMGLIHQETNDCDFTKYFSKGCAPGSEVGSPFCAQCKGSGK
AVGDEYRCKARSEEQYYGYTGAFRCLVEDAGDVAFIKHTIVPESTDGNGPDWAKDLKSSDFEL
LCQDGTTQPVTKFSECHLAKVPAHAVITRPETRGDVVSILLELQAKFGSSGSDSSFRMFQSSVEK
NFLFKDSTKCLQEIPKGTKFQDFLGKEYMIAMQSLRKCSDSTSDLEKACTFHSCQQKE

>*Salmo trutta* (ENSSTUT00000100204.1)

MKLLLVSALLGCLATAYAAPAEGIVRWCVKSEQELRKCHDLAAKVAQFSCVRKDGSFECIQAI
KGGEADAITLDGGDIYTAGLTNYGLQPIIAEDYGEDSDTCYYAVAVAKKGTAFGFNTLRGKKS
CHTGLGKSAGWNIPIGTLVTENQIQWGGIEDRPVESAVSDFFNASCAPGATKGTKLCQLCKGDC
SRSHKEPYYDYAGAFQCLKDGAGDVAFIKPLAVPAAEKASYELLCKDGTRAPIDSYKTCHLAR
VPAHAVVSRKDPELADRIYNKLVAVKDFNLFSSDGYAAKNLMFKDSAQKLVQLPNTTDSFLYL
GAEYMSTIRSLKKSQATGATSRAIKWCAVGHAEKDKCDTWTINSFADGESKIECQNAPTVEECI
KKIMRKEADAITVDGGEVYTAGKCGLVPVMVEQYDADLCSAPGEASSYYAVAVAKKGSGLT
WKTLKGKRSCHTGLGRTAGWNIPMGLIHKETEDCDFTKYFSKGCAPGSEVGSTFCAQCKGSGK
PVGDEDMCKARSEEQYYGYTGAFRCLVEGAGDVAFIKHTIVPESTDGNGPVWAKDLKSSDFEL
LCQDGTTQPVTKFRECHLANVPAHAVITRPETRGDVVSILLEQQAKFGSSGSDSSFKMFQSSVE
KNLLFKDSTKCLQEIPKGTKYQDFLGEEYMIAMQSLRKCSDSTSDLEKACTFHSCQQKE

>*Sander luciop* (ENSSLUT00000055031.1)
MQTLLLVALLGCLAAVFAAPADRVRWCVKSDKEHEKCEALAAKAPAFSCVKKTNTIDCIVAIK
AGEADAITLDGGDIYVAGLKNYDLQPIIAEDYGTSSETCYYAVAVVKKGTEFTIDQLRGKKSCH
TGLGKSAGWNIPIGTLVSMNLIQWRGIEDKPVEEAVSEYFSASCVPGATRSSKLCQLCRGDCSRS
HNEPYYDYGGAFQCLKDGAGDVAFVKHLTVPESEKSNYELLCKDNTRAPIDSYQNCHLAKVP
AHAVVTRKDPQLAQLIWTHLSSVQSLPDFDLFSSEAYAPAKNLMFKDSTLKLVQVPANTDSFLY
LGAEYMSIVRSLKKEPTGTTSTAIKWCAVGHAETAKCDSWSINSVRDDATAAIECARGATVEEC
LKMIMSKEADAMAVDGGQVYTAGKCGLVPAMVEQYESELCSTAGATASSYYAVAVVKRDSG
VTWETLQGKRSCHTGVGRTAGWNIPMGLIHQRTQDCDFTRFFSSGCAPGANSTSPFCTNCRGS
GKAVGDEYKCKASAEEQYYGYAGAFRCLVENAGDVAFIKHTIVSENSDGKNVAPWASGVKSA
DYQLICPGKAPVPVTDYASCHLALVPAHAVVTRPESHGEVVSVLRDQQAKFGSSVPDATFRMF
QSESGKNLLFKDSTKCLQEVQAGKTYADFLGEQYMISMKSLRQCSETTPDLEKSCTFHSCQQKI

>*Scleropages formosus* (ENSSFOT00015048266.1)
MNALLAAAVLLGSLELVASSDQKVKWCLKSEQEKKKCDDLAAKVPRFTCVLRHGSEECIKAIK
EGHADAITLDGGDVYKAGLTNYDLHPIIAEDYGKDSDTCYYAVAVVKKGTGFMWSQLKGKKT
CHTGLGKSAGWNIPVGTLVAQGDITWGGQEDESILKAFSEFVSASCVPGAQKGSNLCQLCKGD
CSRSHREPYYDYDGAFQCLKDGAGDVAFVKHLTVPPSEKAGYELLCKDGTRKDIDEYKSCHLA
RVPAHAVVSRKEPELASRIWDSLQAAQGFPLFSSEGYGAKNLMFKDSTVRLVQLPKTTDSFLYL
GAEYMSTIRSLKKELSSDIQSKAIKWCAVSRVETVKCDTWSIHSVDTGVAKIECQQAPTVDECIK
KIMRKEADAMAVDGGEVYSAGKCGLVPAMVEQYDEDKCGSSNAVASSYYAVAVVKKNSGLT
WTELKEKKSCHTGMGRTAGWNVPMGLIHNQTNDCDFSKFFAKSCAPGADPSSNLCELCVGSG
KAVSGQEHKCKASSEELFYGYAGALRCLVEGDGDVAFMKHTTVKENSGGQGGQWAKDLKPS
DFELLCPSGLAATMPVEQYEKCHLAKVPAHAVVTRPESRQEVVAILKEQQGRFGHSSTAGFKM
FQSKDGKNLLFKDSTKCLQEVPSQQSYEDFLGPEYMASIRSLRQCASSVSDLEQACTFHTCQQK
A

>*Seriola lalandi* dorsalis (ENSSLDT00000015513.1)

MKLLLLVALLGCIAAVFAAPAEKVKWCVKSEQEYRKCLDLAAAAPAFSCVRKENTIDCITAIKA
GEADAITLDGGDIYTAGLTNYNLHPIIAEDYGPTSDTCYYAVAVVKKDTGFGFHDLRGKKTCHT
GLGKSAGWNIPVGTMISMGILPWGGIDDKPVEEAVSEFFLKSCVPGATRPKLCELCKGDCSRSH
KEPYYDYDGAFQCLVEDKGEVAFVKHLTVPESEKANYELLCKDNTRAPISSYKTCNLARVPAH
AVVTRKDPQLAELIWTSLQAVQVKHGFKLFSSEAYAPAKNLMFKDSAEKLVRLPPNIDSFLYLG
AEYMSIVRSLKKEQTPGTSSSAIKWCAVGHAETSKCDQWTINSVGDDGSAIECQNAPSVEECLK
KIMRKEADAMAVDGGQVYTAGKCGLVPVMVEQYDQGQCGNNDAGASSYYAVAVVKKSSGV
TWDTLQGKRSCHTGIGRTAGWNIPMGHIHKRTNDCDFSKFFTSGCAPGADPTSSFCNQCVGSRR
AVGDEYKCKASAEEQYYGYAGAFRCLVENSGDVAFIKHTIVGENSDGNGPAWASGVNSADYQ
LICPGKPPVPVSDFLSCNLAKVPAHAVVTRPESRSEVIRVLQDQQAKFGISGSDASFRIFHSEGGK
NLLFKDSTKCLQEIPSGSNYEQFLGVEYMNAMSSLRQCGETTPDLEKSCTFHSCQQKN

>*Stegastes partitus* (ENSSPAT00000011999.1)
MKLLLCATLLGFLATVLAAPAEKVKWCVKSDAEYQKCLALATAAPAFSCVKKDNTIDCIVAIK
AGDADAITLDGGDVFTAGLNNYDLQPIIAEDYGTTSDTCYYAVAVVKKGSGFGFRDLKGKKSC
HTGLGKSAGWNIPIGTLLTMNILEWAGIEDKPVEEAVSSFFQASCAPGATRGSKLCELCKGDCS
RSHSEPYYDYAGAFQCLVEDAGEVAFVKHLTVPDAEKSKYELLCKDNSRAPIDNYKTCNLARV
PAHAVVTRKDAQLAEFIWTSLNGVQGFNLFSSEGYTGKNLMFKDSTTKLVKLPPNTDSFLYLG
AEYMSVLRSLKKEQLPGTVSPAIKWCAVGHAETVKCDTWSINSVDGENAVIECQSAPTVDECL
KKIMRKEADAMAVDGGQVYTAGKCGLVPVMVEQYENNLCSSSEGVASSYYAVAVVKKSSGV
TWETLQGKKSCHTGMGRTAGWNIPMGRIYERTKDCDFTKFFSSGCAPGADANSPFCSQCAGSG
KAVGDEAKCKASADEQYYGYAGAFRCLVEGAGDVAFIKHTIVKENSDGVGPAWASAVSSGDY
QLICPGKEPAEITDFANCHLAIVPAHAVVTRPESRADVIRILQDQQAKFGSSGSDPTFKLFQSDQG
KNLLFKDSTQCLQEVAAGKSYGQFLGPEYTNAMASLRHCSDSTPDLEKSCTFHSCQQA

>*Takifugu rubripes* (ENSTRUT00000071311.1)
MLDSALKLDFQMLCEMCDQRGVSIATTFAPALGNADSANTTARTSSMKTLLLAVLLGCLAAAS
AVPAARIRWCLKSEAEYLKCKRLELVAPAISCVRRESTMECIVAITAKLADAITLDGGDVYTAG
LKNYDLHPIIAEDYGPSSDTCYYAVAVVKKGSSFGIKDLAGKKSCHTGLGKSAGWNIPIGTLLS
MDLIKWTGIEDSPVEEAVKNFFHSSCVPGANANDKLCQLCKGDCSRSHKEPYYDYAGAFQCLA
DGAGEVAFVKHLTVPESEKPSYELLCPDNTRKSIDSYKTCHLARVPAHAVVSRKDPQMAELIYN
TLTTVRGFNLFSSEDYAPAQNLMFKDSTIRLVKLPPNTDSFLYLGAGYMSIIRSLKREQATSAAP
TAIKWCAVGPAETAKCDTWSINSIEGEVTNVECHSAKSVEDCMSMIMRKRADAMAVDGGQVY
TAGKCGLVPVMVEQYDEAQCSVSSAPASSYYAVAVVKKGMGITWETLKGKRSCHTGMGRTA
GWNIPMGLIHKQTNNCDFTTFFSSGCAPGAEPTSPFCAACAGSSKSVGDEYKCKPSAEEHYYGY
AGAFRCLVEGAGDVAFIKHTIVKENSDGNGPDWARNVNSADYELICPNKSPVPVTDFASCHLA
MVPAHAVVTRPESRGDVVRILQDQQAKFGTKGTDGRFKLFQSESGKNLLFKDSTKCLQEIPLGE
SFEKFLGAEYMTAMSSLRVCTDSTPDLEKICTFHSCQQKS

>*Tetraodon nigroviridis* (ENSTNIT00000018522.1)

MKTLLLAALLGCLVVAFAAPTDRVRWCLKSEQEHVKCKRLAAVAPAITCVPKQSTLDCIVAIK
DGLADAITLDGGDIYTAGLNNYDLHPIIAEDYGPSSETCYYAVAVVKKGSGFGIRDLAGKKSCH
TGLGKSAGWNIPIGTLLSMGLIQWSGIEDSPVEEAVKNYFQSSCVPGAKPGSKLCQLCKGDCSR
SHKEPYYDYSGAFQCLADGVGDVAFVKHLTVPDSEKSKYELLCRDGTRKPIDSYETCHLARVP
AHAVVSRKDPQLADLIYTTLTTIRGFSLFSSEDYAPAKDLIFKDSTTQLVKLPPKTDSFLYLGAEY
MSIIRSLKREQALSATPSAVKWCAVGPAETEKCDTWSINSIDNDVTTIECQTAPSVEECIKMIMR
KEADAIAVDGGQVYTAGKCGLVPVIAEQYDDAQCSAPSATTSSYYAVAVVKKGAGITWDTLK
GKKSCHTGVGRTAGWNIPMGLI

>*Xiphophorus couchianus* (ENSXCOT00000019341.1)
MKTLLLVVLLGCLAAVFAAPAAKVRWCVKSDQELRKCTDLAAAAPEFSCVKKESTLECIIAIKA
SEADAITVDGGDVYTAGLNNYDLHPILAEDYGETSDTCYYAVAVVKKGTNFGIRDLGGKKSCH
TGLGKSAGWNIPIGTLISMNVIQWAGIEDKPVEEAVSTFFQASCAPGATRGSKLCELCKGDCSRS
QREPYYDYSGAFQCLAEDAGEVAFVKHLTVPDSEKSKYELLCLDNSRAPIDSYKTCHLARVPA
HAVITRKDPALAELIRNSLSGVQNFNLFSSEAYAPAKDLMFKDSAQRLVPVPPNTDSFLYLGAD
YVSIIHSLRKEQSTGSESASIRWCAVGHAETAKCDTWSINSVTDDTASVECQNAPTVEDCFKKIM
RKEADAVAVDGGQVYTAGKCGLVPAMVEQYQQELCGSSPDTASSYYAVAVVKKSSGVTWAN
LAGKKSCHTGVGRTAGWNIPMGHIYAQTKDCNFANFFSSGCAPGAEANSPFCRECKGSGKAVG
DEAKCKASAEEQYYGYAGAFRCLVEGAGDVAFIKHSIVSENSDGNGPDWARGVNSDDYQLICP
GKDPVPVGGFASCHLAVVPAHAVVTRPDVRDKVVRILQDQQTKFGASGSDPTFRMFQSANGK
NLLFKDSTKCLQEVSQGKSYEQFLGPEYINAMSSLRQCSDTASDLEKSCTFHTCQQP

>*Xiphophorus maculatus* (ENSXMAT00000016142.2)
MWKLRSDLCFLVYQHTKVHRRPHIRKPEQTVVFYLLASSGRLCYKSHNPVTESLLCPIGQETPQ
STSMKTLLLVVLLGCLAAVFAAPAAKVRWCVKSDQELRKCTDLAAAAPEFSCVKKESTLECIIA
IKASEADAITVDGGDVYTAGLNNYDLHPILAEDYGETSDTCYYAVAVVKKGTNFGIRDLGGKK
SCHTGLGKSAGWNIPIGTLISMNVIQWNGIEDKPVEEAVSTFFQASCAPGATRGSKLCELCKGDC
SRSQREPYYDYSGAFQCLADDAGEVAFVKHLTVPDSEKSKYELLCLDNSRAPIDSYKTCHLAR
VPAHAVITRKDPALAELIRNSLSGVQNFNLFSSEAYAPAKDLMFKDSAQRLVPVPPNTDSFLYL
GADYVSIIHSLRKEQSTGSESSSIRWCAVGHAETAKCDTWSINSVTDDTASVECQNAPTVEDCF
KKIMRKEADAVAVDGGQVYTAGKCGLVPAMVEQYQQELCGSSPDTASSYYAVAVVKKSSGV
TWANLAGKKSCHTGVGRTAGWNIPMGHIYAQTKDCNFANFFSSGCAPGAEANSPFCRECKGS
GKAVGDEAKCKASAEEQYYGYAGAFRCLVEGAGDVAFIKHSIVSENSDGNGPDWARGVNSDD
YQLICPGKDPVPVGGFASCHLAVVPAHAVVTRPDVRDKVVRILQDQQTKFGASGSDPTFRMFQ
SANGKNLLFKDSTKCLQEVSQGKSYEQFLGPEYINAMSSLRQCSDTASDLEKSCTFHTCQQP
